# Engineering threshold-based selection systems

**DOI:** 10.1093/g3journal/jkab234

**Published:** 2021-07-14

**Authors:** Katherine H Pedone, Vanessa González-Pérez, Luciana E Leopold, Neal R Rasmussen, Channing J Der, Adrienne D Cox, Shawn Ahmed, David J Reiner

**Affiliations:** 1 Lineberger Comprehensive Cancer Center, University of North Carolina, Chapel Hill, NC 27599, USA; 2 Curriculum in Genetics and Molecular Biology, University of North Carolina, Chapel Hill, NC 27599, USA; 3 Department of Biology, University of North Carolina, Chapel Hill, NC 27599, USA; 4 Institute of Biosciences and Technology, College of Medicine, Texas A&M Health Science Center, Houston, TX 77030, USA; 5 Department of Pharmacology, University of North Carolina, Chapel Hill, NC 27599, USA; 6 Department of Radiation Oncology, University of North Carolina, Chapel Hill, NC 27599, USA

**Keywords:** EGL-1, BH3-only, SMG-1, nonsense-mediated decay, NMD, 3ʹUTR, small GTPase, EHT 1864, EHT 8560, Pak

## Abstract

Using model organisms to identify novel therapeutic targets is frequently constrained by pre-existing genetic toolkits. To expedite positive selection for identification of novel downstream effectors, we engineered conditional expression of activated CED-10/Rac to disrupt *Caenorhabditis elegans* embryonic morphogenesis, titrated to 100% lethality. The strategy of engineering thresholds for positive selection using experimental animals was validated with pharmacological and genetic suppression and is generalizable to diverse molecular processes and experimental systems.

## Introduction

Harnessing model invertebrates to screen for small-molecule inhibitors or for new genetic components of known processes is desirable because of the phylogenetic conservation of many key metazoan proteins and the infrequency of gene redundancy compared to mammals. Direct, unbiased screening for phenotypes is potentially powerful. Yet direct screening, whether with libraries of small molecules, RNAi, or chemical mutagenesis, can be difficult due to extensive phenotypic buffering and nonhomolog redundancy in many biological processes. Additionally, potential screens depend on the availability of optimally suited reagents and/or phenotypes. These tools are frequently missing.

Screens can also demand sensitivity. For example, small-molecule inhibitors must confer robust phenotypes, which may thus exclude potentially valuable lead compounds that, in their initial formulation, would confer modest effects in initial screens. Screens can also be very laborious, particularly when scaled up to levels of throughput necessary to detect rare positive candidates that confer incompletely penetrant or expressive phenotypes. Engineering of sensitized platforms that allow for the identification of even atypical or rare candidates will help revolutionize approaches to contemporary experimental biology.

We have devised a method for engineering sensitized screening platforms using the experimental model organism *Caenorhabditis* *elegans*. This scheme is theoretically generalizable to any experimental organism. Our inspiration came from the activating mutation *let-60(n1046*gf*)* in the *C. elegans* ortholog of the human RAS oncoprotein. This G13E gain-of-function mutation induces ectopic 1˚ vulval cells and hence a 100% penetrant Multivulva phenotype that is exquisitely sensitive to perturbation of downstream genes (Beitel *et al.* 1990; Han and Sternberg 1990), including genes that, when mutated alone, do not cause strong phenotypes, due to modulatory roles or redundancy (Kornfeld *et al.* 1995; Sundaram and Han 1995; Selfors *et al.* 1998; Sieburth *et al.* 1998). Consequently, we aimed to develop a system where reagents similar to *let-60(n1046*gf*)* could be engineered on demand and then exploited for genetic and pharmacological discovery screens.

## Materials and methods

### Strains and animal handling

Animals were cultured as described (Brenner 1974) and handled in the default 20°C incubator if not otherwise noted, or in dedicated 15°C, 23°C, or 25°C incubators. T-curves were performed in an incubator changed stepwise for each temperature. For conditional hypodermal assays, parental strains were grown at 15°C. Parents were shifted to the assay temperature at the late L4 stage and their progeny were evaluated, including for drug assays. For locomotion assays of *smg-1* mutants, strains were maintained continually at each assayed temperature and assayed in parallel on the same days. Strains used in this study are presented in Supplementary Table S3.

### Microscopy

Animals were mounted in 2 mM tetramisole/M9 buffer on slides with agar pads. Animals were imaged with a Nikon Eclipse TE2000U microscope equipped with DIC optics, 40×, 60×, and 100× oil objectives, with a DVC-1412 CCD camera (Digital Video Camera Company) controlled by Hamamatsu SimplePCI acquisition software. Some animal handling and imaging were performed on a Leica stereofluorescence microscope equipped with automated zoom optics. For imaging of the *smg-1(*ts*)*; *reIs8[P_lin-26_::gfp]* strain, mixed stage embryos were mounted live in M9 buffer on slides with a 3% agar pad. DIC/Nomarski optics and fluorescence microscopy were captured using a CSU-W1 spinning-disc confocal laser microscope with 405, 488 nm lasers and Photometrics Prime BSI camera. Captured images were then processed using NIS Elements Advanced research, version 4.40 (Nikon).

### Small-molecule treatment

Pharmacological treatment with EHT 1864 and inactive analog EHT 8560 (provided by Virginie Picard, Exon Hit Therapeutics) was performed in 6-well microtiter plates with small volumes of agar, with only corner wells used. EHT 1864 was diluted to the appropriate concentration in 1% DMSO, with 1% DMSO without inhibitor as a control. Animals were grown in an incubator dedicated to 23°C, with all animals for a given assay grown in parallel.

### Locomotion assay

Animals were subjected to a circumferential locomotion assay as described previously (Reiner *et al.* 2006). Briefly, young adult animals were picked to the center of a 10-cm plate seeded 1 day previously, the origin was marked on the bottom, and animals were allowed to roam freely for 20 min, at which point they were arrested by placing at −20°C for 5 min. The distance from the origin was measured for each animal.

### Molecular biology

Details of plasmid construction and PCR detection of mutations are available upon request. Primers used in this study are presented in Supplementary Table S4. Plasmids used are presented in Supplementary Table S5.

## Results and discussion

**Figure 1:**
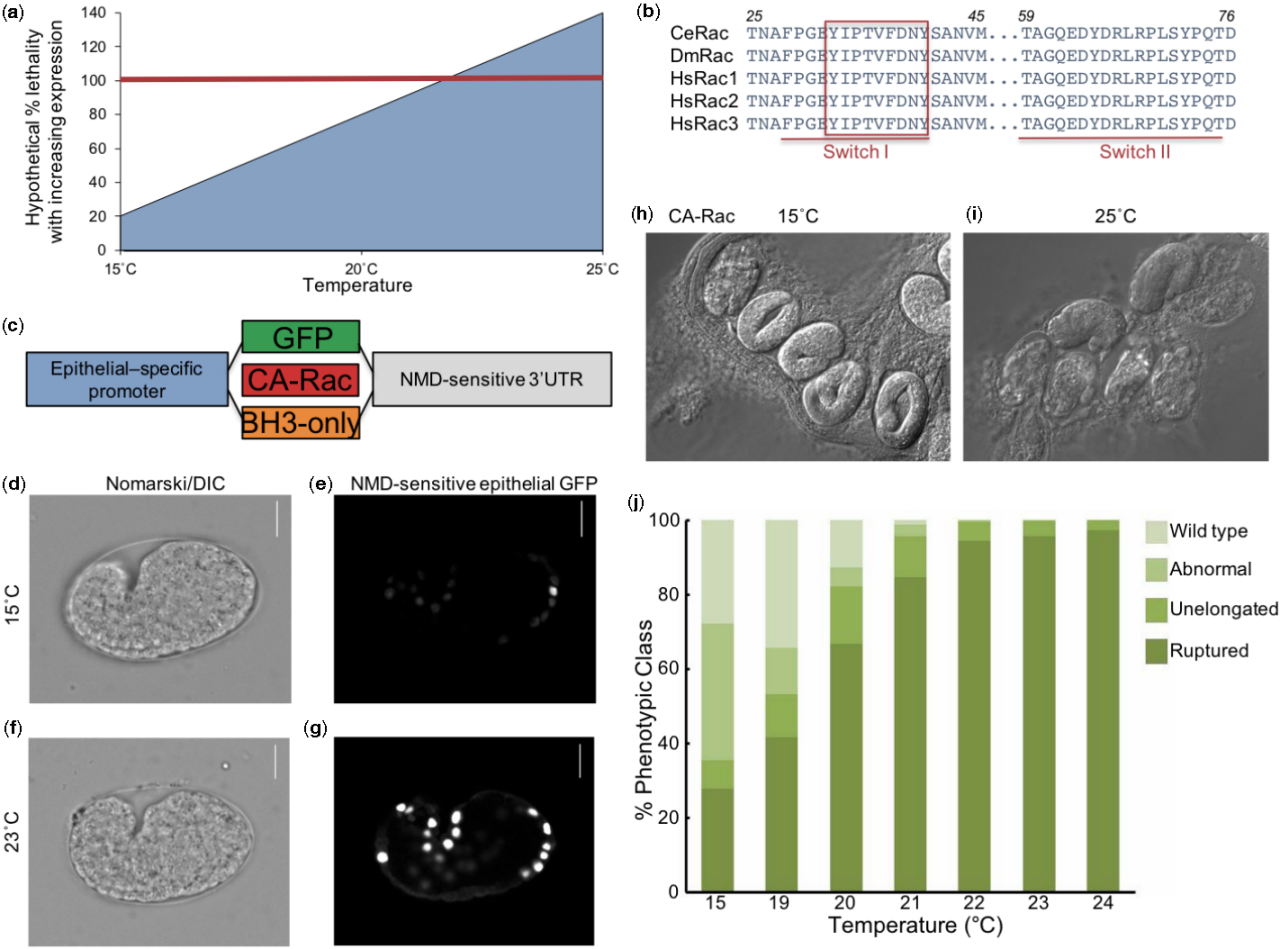
A system for conditional expression of signaling proteins to titrate to a threshold of 100% defective. (A) A hypothetical graph of temperature-controlled levels of gene product required to reach the threshold of 100% lethality. (B) One hundred percent residue identity among Rac GTPases of *C. elegans*, *Drosophila melanogaster*, and humans in the structurally critical Switch I and II regions that harbor the core effector-binding sequence (boxed). (C) A schematic of plasmids for conditional expression of proteins, either control GFP, constitutively activated CED-10/Rac, or proapoptotic BH3-only protein EGL-1. The promoter is the “eFGHi” variant of the *lin-26* promoter, which drives expression in hypodermis (epithelial) cells in the embryo; the NMD-sensitive 3ʹUTR is inverted coding sequences from the *let-858* gene (A. Fire, personal communication). (D–G) Temperature control of epithelial-specific expression from integrated transgene *reIs8* of GFP in epithelial cells under control of the hypodermal promoter and NMD-sensitive 3ʹUTR, in an *smg-1(cc546*ts*)* mutant background for temperature-sensitive perturbation of NMD. (D, E) 100× DIC and epifluorescence micrographs, respectively, of a medial section of an enclosing embryo grown at 15°C reveals epithelial-specific expression and leakiness in the expression system. (F, G) 100× DIC and epifluorescence micrographs, respectively, of medial sections of enclosed (center) and earlier stage (right) embryos grown at 23°C demonstrate elevated temperature-specific expression in epithelial cells and the absence of expression in earlier embryos, when *lin-26* expression is not activated and epithelial fate remains unspecified. Scale bars = 10 µm. See Supplementary Figure S1 for hypodermal expression in different focal planes. (H, I) 60× DIC images of *reIs6* animals expressing constitutively activated CA-Rac^CED-10^ at 15°C and 23°C, respectively. (H) Animals grown at 15°C show a mixture of stages or hatched L1, and (I) animals picked after growth for 24 hours at 25°C show 100% rupture or arrested elongation. (J) A curve of animal defects and survival at stepped temperatures from 15°C to 24°C. Animals were binned into different classes based on morphology. “Abnormal” = observed lumps on the surface of hatched animals. “Unelongated” = intact embryos that failed to elongate. “Ruptured” = embryos that failed enclosure and so therefore exploded.

We started with a signal known from mammalian studies and well conserved in *C. elegans*, but where the optimal genetic tools have not been previously generated by the research community. We used a conditional expression system to express potentially toxic protein whose full activation confers 100% lethality. As needed, lethality could theoretically be conferred either at the level of failure of essential multicellular processes or the viability of single cells. This lethality thus establishes a threshold for positive selection for identifying suppressing compounds or mutations in the process of interest (Figure 1A).

*C*aenorhabditis *elegans* Rac^CED-10^ is identical to human Rac in the critical Switch I and II regions involved in effector and regulator interactions (Figure 1B). We focused specifically on essential morphogenetic functions of CED-10 that occur in differentiated cells that are postmitotic, thus avoiding potentially complicated analyses of multiple biological processes in parallel (Chisholm and Hardin 2005).

To express proteins specifically in hypodermal (epithelial) cells, we expressed cDNAs encoding green fluorescent protein (GFP) or potentially toxic proteins behind the *eFGHi* variant of the *lin-26* promoter (Figure 1C;Supplementary Figure S1; Landmann *et al.* 2004). To achieve conditional expression, we used an aberrant synthetic 3ʹUTR that is subject to nonsense-mediated mRNA decay (NMD); in a wild-type background, the expressed mRNA in predicted to be degraded. Transgenes were then expressed in a temperature-sensitive (ts) mutant for *smg-1*, an essential component of the NMD process. Thus, at permissive temperature of 15°C, NMD is predicted to be functional, the expressed mRNA with the aberrant synthetic 3ʹUTR is detected, and the heterologously expressed mRNA should be degraded. At restrictive temperature of 25°C, NMD is predicted to be nonfunctional, the expressed mRNA with the aberrant synthetic 3ʹUTR should not be detected, and the heterologously expressed mRNA should be stable, thereby permitting full expression of GFP or toxic protein. This phenomenon may be titratable with temperature.

We initially became interested in NMD by identification of a mutation in the gene *unc-97* that was suppressible by disruption of NMD via null (*re1* and *re861*) or temperature-sensitive (*cc545* and *cc546*) mutations in SMG-1, a conserved protein with a domain similar to that of PI3 Kinase. We confirmed the temperature-sensitivity of *smg-1* alleles *cc545* or *cc546* alleles using behavioral and semiquantitative RT-PCR experiments for *unc-54(r293)*, which harbors a mutation in the 3ʹUTR of the endogenous *unc-54* gene that confers NMD-dependent loss of function and defective locomotion (Supplementary Figures S2–S5). We found that *cc545* and *cc546* are predicted to cause single amino acid changes in conserved residues in the SMG1 and PI3Kc domain, respectively (Supplementary Figures S6 and S7), consistent with the hypothesis that temperature-sensitive mutations perturb protein structure or stability of SMG-1 in a manner that could allow for temperature-sensitive regulation of NMD.

We engineered expression of GFP cDNA under the control of a synthetic NMD-sensitive (NMD^S^) 3ʹUTR, all in the *smg-1(cc546*ts*)* genetic background. At restrictive temperature, where NMD is inactivated and hence mRNA stabilized and protein expressed, we observed high levels of GFP in embryonic epithelia. At the permissive temperature, where NMD is functional, we observed lower levels of GFP, revealing some leakiness in NMD-dependent degradation of experimental mRNA (Figure 1, D–G; Supplementary Figure S1). Conditionally expressed GFP did not induce embryonic lethality, though weak morphological dysgenesis was observed (Supplementary Table S1 and Figure S8). Thus, expression was temperature-sensitive, restricted to embryonic epithelial cells, and nontoxic.

To test our hypothesis that titratable expression of toxic proteins could be regulated with the NMD^S^ 3ʹUTR, we generated transgenic animals expressing Q61L mutant (constitutively active) CA-Rac^CED-10^. To achieve 100% activation only in the hypodermal cells, we chose constitutive activation of Rac^CED-10^ over deletion of negative regulatory GAPs that stimulate hydrolysis of GTP and hence inactivation of Rho family small GTPases, including Rac. Deletion of GAPs would be expected to derepress endogenous Rac^CED-10^ in some subcellular compartments but not others and contend with likely redundancy between multiple GAPs, action in multiple tissues in the animal, and substrate cross-specificity with related small GTPases like RHO-1, CDC-42, RhoG^MIG-2^, and others.

We observed that CA-Rac^CED-10^-dependent embryonic lethality was titratable based on 1-degree steps of temperature. At restrictive temperatures, we observed failure of embryonic morphogenesis and elongation, with 100% embryonic lethality at 22°C. We decided to focus on 23°C as a fully penetrant nonpermissive temperature for future experiments (Figure 1, H–J). Thus, our system is capable of generating conditional lethality calibrated to 100%.

To further validate our expression system using a different signaling modality, we expressed the proapoptotic BH3-only protein, EGL-1 (Conradt and Horvitz 1998). At restrictive temperature, this transgene induced ∼100% lethality, was weakly suppressed by CED-3/caspase-directed RNAi, and strongly suppressed by the mutation *ced-3(n717)* in the *C. elegans* caspase (Supplementary Table S2). At the permissive temperature, little or no effect of this transgene was observed, thereby corroborating the threshold-based selection paradigm we sought. This temperature-sensitive NMD-sensitive system has also been harnessed for conditional expression of toxic signaling proteins for purposes of interrogating biological functions (Walck-Shannon *et al.*^2015^, ^2016^).

**Figure 2:**
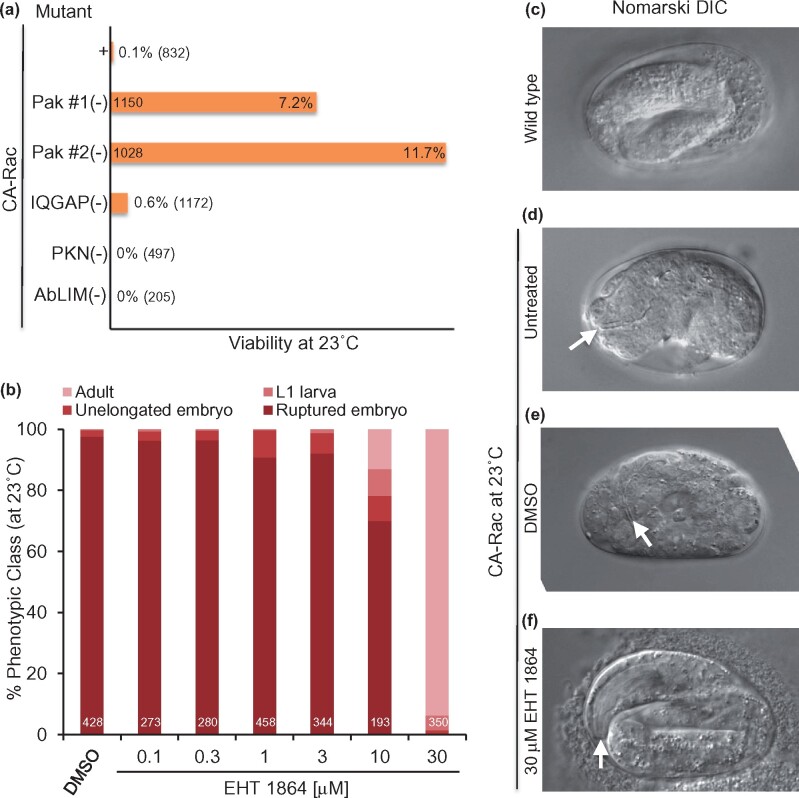
Genetic and pharmacological blockade of constitutively activated CED-10/Rac. (A) In the *smg-1(cc546*ts*)*; *reIs6[Plin-26::ced-10(Q61L)::NMD^S^3*ʹ*UTR]* background, different mutations reduced levels of lethality. Two independent strain constructions with the *max-2(nv162)* mutation in the known Rac effector Pak suppressed lethality. Not shown is a synthetic lethal phenotype of *smg-1(cc546*ts*)*; *reIs6* in combination with disruption of the other Pak ortholog, *pak-1(ok448)*. We constructed a strain with *pak-1(ok448)* as a heterozygote but could not homozygose the *pak-1* mutant chromosome. Unlike MAX-2/Pak, PAK-1/Pak has been implicated as an effector of both CED-10/Rac and CDC-42/Cdc42, as well as a GTPase- and kinase-independent component of the Pak-Pix-Git1 complex that regulates the cytoskeleton (Hoefen and Berk 2006; Lucanic *et al.* 2006; Peters *et al.* 2013). Thus, disruption of PAK-1, unlike disruption of MAX-2, is expected to impact multiple signaling systems, perhaps explaining the synthetic lethality observed when PAK-1 is deleted in the CA-CED-10/Rac transgenic background at any temperature. A putative null mutation in PES-7/IQGAP, *pes-7(gk123)*, a putative Rac effector identified in mammalian studies (Watanabe *et al.* 2015), weakly suppressed lethality, while mutations in other Rac effectors PKN-1/PKN (*pkn-1(ok1673)*) (Lu and Settleman 1999) and UNC-115/AbLIM (*unc-115(e2225)*) (Struckhoff and Lundquist 2003) failed to suppress. (B) Rescue of toxicity of constitutively active CED-10/Rac at higher concentrations of the Rac inhibitor, EHT 1864. Classes of phenotypes were binned as in Figure 1. (C) DIC image of an embryo conditionally expressing GFP at 23°C. The lumen of the pharynx is out of the plane of focus, facing left. (D) DIC image of a ruptured embryo conditionally expressing CA-Rac/CED-10 at 23°C. The lumen of the intact pharynx is in focus and facing left (white arrowhead), indicating that development persists even when epithelial morphogenesis is disrupted. (E) DIC image of an embryo conditionally expressing constitutively activated Rac/CED-10 at 23°C and grown on 1% DMSO. The lumen of the intact pharynx is in focus and facing downward (white arrowhead). (F) DIC image of an embryo conditionally expressing constitutively activated Rac/CED-10 at 23°C and grown on 30 µM EHT 1864 in 1% DMSO.

We validated our screening methodology with mutations in Rac effectors and a selective small-molecule inhibitor of mammalian Rac. The Pak serine/threonine kinase is a classic Rac effector that controls cytoskeletal dynamics and contributes to morphogenetic events downstream of Rac^CED-10^ in *C. elegans* (Lucanic *et al.* 2006; Peters *et al.* 2013). Mutation of Pak^MAX-2^ partially suppressed CA-Rac^CED-10^-dependent lethality (Figure 2A). Mutation of other known effectors was mostly inconsequential. Our genetic data indicate that CA-Rac^CED-10^ signals through at least one known effector.

An interesting question is to ask whether we could uncover essential genes in mutant screens to suppress CA-Rac^CED-10^-dependent lethality. We hypothesize that this approach would succeed, but through identification of reduction-of-function or domain-specific alleles rather than the total abrogation of gene/protein function.

We and others showed previously that the small molecule EHT 1864 blocked Rac effector signaling and induction of membrane ruffling in mammalian cells (Desire *et al.* 2005; Shutes *et al.* 2007). As overexpression of activated small GTPases like Rac could have unintended effects on cell biology, we asked if a selective inhibitor of Rac could suppress the effects of CA-Rac^CED-10^-dependent lethality. Treatment with EHT 1864 completely reversed the lethality conferred by CA-Rac^CED-10^ (Figure 2, B–F; Supplementary Figure S9). Most rescued animals appeared normal. Wild-type animals exposed to the same dose-response curve did not exhibit increased lethality. The structurally related negative control molecule EHT 8560 failed to rescue. These results affirm that it is CA-Rac^CED-10^ that conferred specific embryonic lethality and that compounds capable of inhibiting mammalian Rac can function to suppress Rac^CED-10^ activity in a distinct metazoan. Consequently, this screening platform could be used for the identification of lead compounds to inhibit specific “pathways.” For greater specificity, protein targets with less conservation at the sequence level could be humanized to improve the odds of successful translation to mammals.

In summary, we have shown that it is possible to engineer specific biochemical pathways to establish the key threshold of 100% lethality. Such engineering can thereby selectively sensitize these pathways to both genetic and/or chemical suppression, via principles derived from classical suppressor genetics. We expect that the CRISPR revolution will further expand the flexibility, power, and sensitivity of such engineered thresholds. We speculate that if the appropriately sensitive tissue can be defined, this approach is generalizable to any experimental organism or biological process, including to humanized targets where this is advantageous. Conventional targeted therapies are based on the assumption that researchers have identified the best pharmacological target. A strength of our approach is that genetically sensitizing pathways in a model organism employ a different set of assumptions, and so lets the biology tell us which targets are most important for function. Thus, our study provides a paradigm for directed selection efforts targeting diverse pathways and various experimental genetic organisms in a manner that is broadly applicable in experimental biology.

## Supplementary Material

jkab234_Supplementary_Data
