# Supplementary material for: Engineering threshold-based selection systems
Source: G3 (Bethesda). 2021 Jul 14;11(9):jkab234. doi: 10.1093/g3journal/jkab234 (PMC8496214; doi:10.1093/g3journal/jkab234)
Supplement: jkab234_Supplementary_Data [file jkab234_supplementary_data.zip › jkab234-suppl_data/GENETICS-G3-2021-402425-s01.pdf]

Supplementary information for

Engineering Threshold-Based Selection Systems

Katherine H. Pedone, Vanessa González-Pérez, Luciana E. Leopold, Neal R Rasmussen, Channing J. Der, Adrienne D. Cox, Shawn Ahmed and David J. Reiner

CONTENTS **page**

Supplementary Methods	2-3
Supplementary Figure 1: Epithelial-specific GFP expression at 15˚C	4
Supplementary Figure 2: NMD-dependent differences in gene expression	5
Supplementary Figure 3: A *smg-1* temperature-sensitive allele regulates locomotion	6
Supplementary Figure 4: TS NMD-sensitive *unc-97(su110)*.	7
Supplementary Figure 5: Quantification of NMD-dependent differences on locomotion	8
Supplementary Figure 6: Identification of lesions in *smg-1*	9
Supplementary Table 1: Lethality conferred by component reagents	10
Supplementary Figure 7: Alignment of worm, fly and humans SMG-1 orthologs	11-17
Supplementary Figure 8: Weak morphogenetic phenotypes	18
Supplementary Table 2: EGL-1/BH3-only-induced lethality is caspase-dependent	19
Supplementary Figure 9: EHT 1864 rescue vs. negative control EHT 8560	20
Supplementary Table 3: *C. elegans* strains used in this study	21
Supplementary Table 5: Plasmids used in this study	22
Supplementary Table 4: Primers used in this study	23
Supplementary protocol for generating toxic transgenes	24-29
Supplementary figure legends	30-33
Supplementary Bibliography	34-35

Supplementary Methods

Strains and animal handling. Animals were cultured as described[1] and handled in the default 20˚C incubator if not otherwise noted, or in dedicated 15˚C, 23˚C or 25˚C incubators. T-curves were performed in an incubator changed stepwise for each temperature. For conditional hypodermal assays, parental strains were grown at 15˚C. Parents were shifted to the assay temperature at the late L4 stage and their progeny were evaluated, including for drug assays. For locomotion assays of *smg-1* mutants, strains were maintained continually at each assayed temperature and assayed in parallel on the same days. Strains used in this study are presented in **Supplementary Table 3**.

Microscopy. Animals were mounted in 2 mM tetramisole/M9 buffer on slides with agar pads. Animals were imaged with a Nikon Eclipse TE2000U microscope equipped with DIC optics, 40x, 60x and 100x oil objectives, with a DVC-1412 CCD camera (Digital Video Camera Company) controlled by Hamamatsu SimplePCI acquisition software. Some animal handling and imaging was performed on a Leica stereofluorescence microscope equipped with automated zoom optics. For imaging of the *smg-1(*ts*)*; *reIs8[P_{lin-26}::gfp]* strain, mixed stage embryos were mounted live in M9 buffer on slides with a 3% agar pad. DIC/Nomarski optics and fluorescence microscopy were captured using a CSU-W1 spinning-disc confocal laser microscope with 405, 488nm lasers and Photometrics Prime BSI camera. Captured images were then processed using NIS Elements Advanced research, version 4.40 (Nikon).

Small molecule treatment. Pharmacological treatment with EHT 1864 and inactive analog EHT 8560 (provided by Virginie Picard, Exon Hit Therapeutics) was performed in 6-well microtiter plates with small volumes of agar, with only corner wells used. EHT 1864 was diluted to the appropriate concentration in 1%

DMSO, with 1% DMSO without inhibitor as a control. Animals were grown in an incubator dedicated to 23˚C, with all animals for a given assay grown in parallel.

Locomotion assay. Animals were subjected to a circumferential locomotion assay as described previously[2]. Briefly, young adult animals were picked to the center of a 10 cm plate seeded 1 day previously, the origin was marked on the bottom, and animals were allowed to roam freely for 20 min, at which point they were arrested by placing at -20˚C for 5 min. The distance from the origin was measured for each animal.

Molecular biology. Details of plasmid construction and PCR detection of mutations is available upon request. Primers used in this study are presented in **Supplementary Table 4**. Plasmids used are presented in **Supplementary Table 5**.

Pedone Supplementary Fig. 1

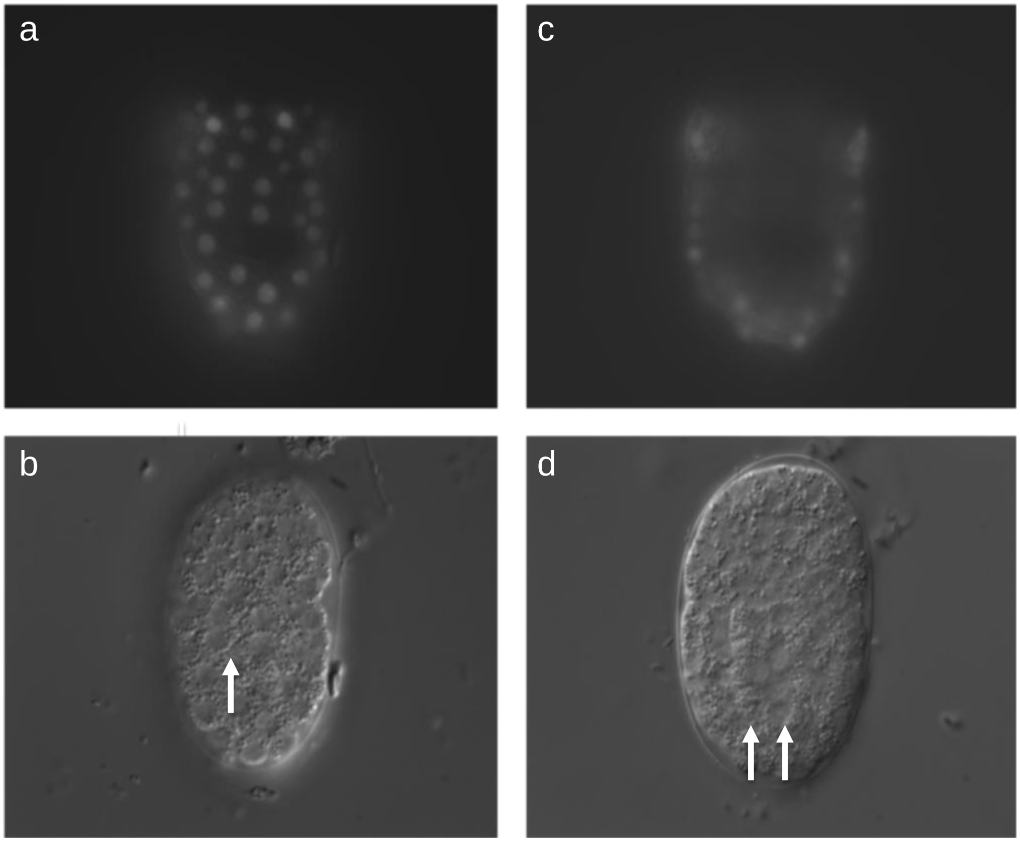

Supplementary Figure 1. Epithelial-specific GFP expression at 15˚C. a-d) The same early enclosure staged *smg-1(cc546*ts*)*; *reIs8[P*$_{lin-26}$*::gfp::NMD*^{S}*3'UTR]* embryo in different focal planes. **a, b)** Epifluorescence (500 msec exposure) and DIC images, respectively, of the dorsal surface of the embryo, with arrows indicating a row of intercalating epithelial cells. **c,d)** Epifluorescence (500 msec exposure) and DIC images, respectively, of a medial section of the embryo, with arrows indicating the column of intestinal cells.

Pedone Supplementary Fig. 2

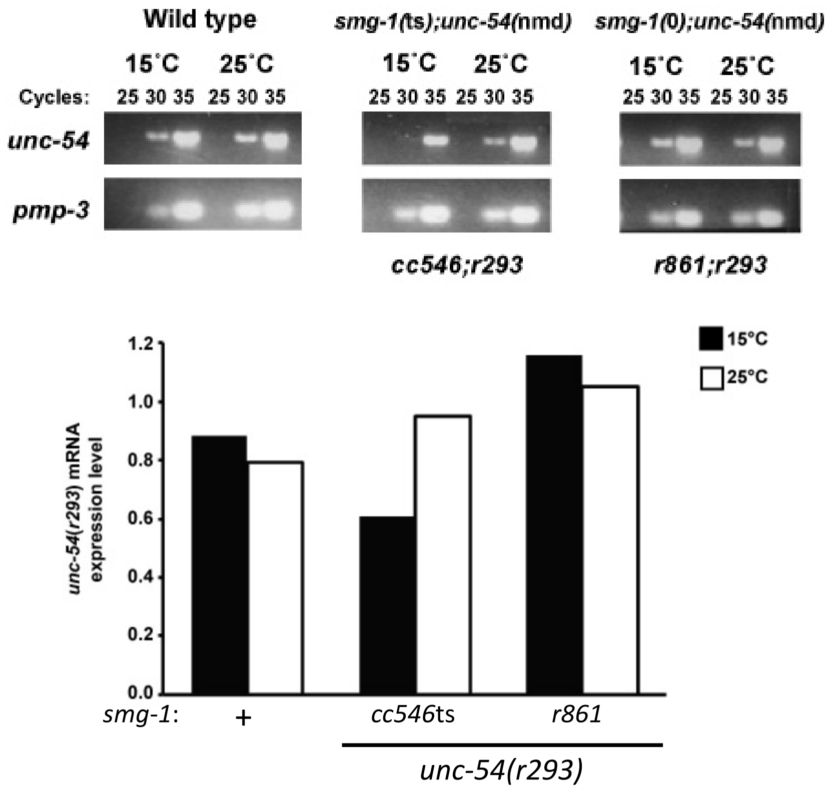

Supplementary Figure 2. NMD-dependent differences in gene expression. Animals harboring the *cc546* temperature-sensitive mutation in *smg-1* have increased *unc-54(r293)* mRNA levels at 25°C by RT-PCR, with *pmp-3* RNA as a control. RNA extractions were performed on pools of adult animals raised at either 15°C or 25°C. cDNA preparations of each strain were subjected to 25, 30 or 35 cycles of PCR with *unc-54*-specific primers. Temperature-dependent differences were visible at 30 cycles with the *cc546*ts allele of *smg-1* used in this study but not the *smg-1(+)* or *smg-1(r861)* putative null mutation, as shown in the graph. Band intensities were quantified using the Image J gel analysis tool. Experiment was performed two times.

Pedone Supplementary Fig. 3

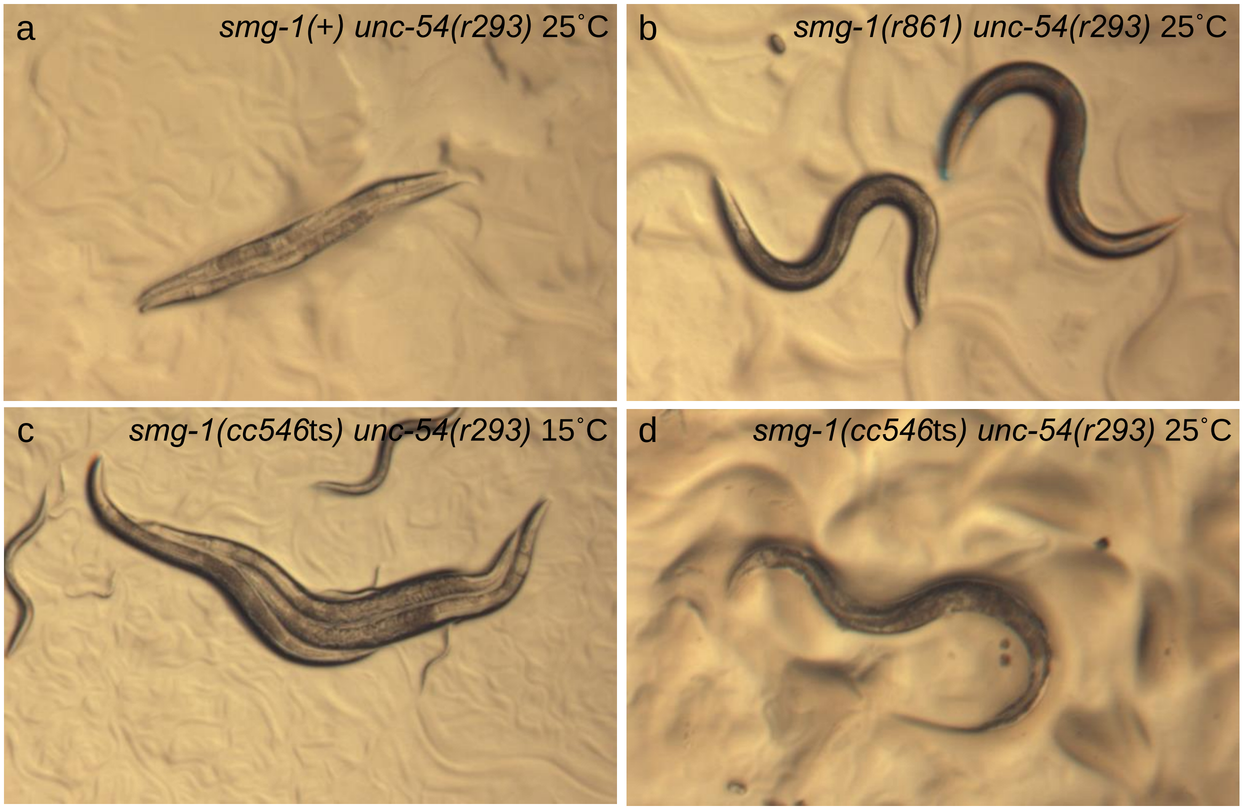

Supplementary Figure 3: A *smg-1* temperature-sensitive allele regulates locomotion. All strain backgrounds harbor NMD-sensitive *unc-54(r293*). Photomicrographs were captured from agar plates with 25 msec exposures under same lamp settings. Body posture is representative of locomotion and hence myosin production by the *unc-54* gene and its NMD-sensitive mutation in the *unc-54* 3'UTR, *r293*: deep body bends represent typical locomotion, shallow bends represent flaccid paralysis. **a)** *unc-54(r293)* animals were paralyzed and egg-laying defective (Egl). **b)** The locomotion and Egl defects of the *r293* mutant were strongly rescued by loss of *smg-1* function. **c)** Locomotion and Egl defects were not as severe with *cc546*ts as with *smg-1(+)* at 15˚C and **d)** are completely suppressed at 25˚C, consistent with *cc546*ts being temperature sensitive.

Pedone Supplementary Fig. 4

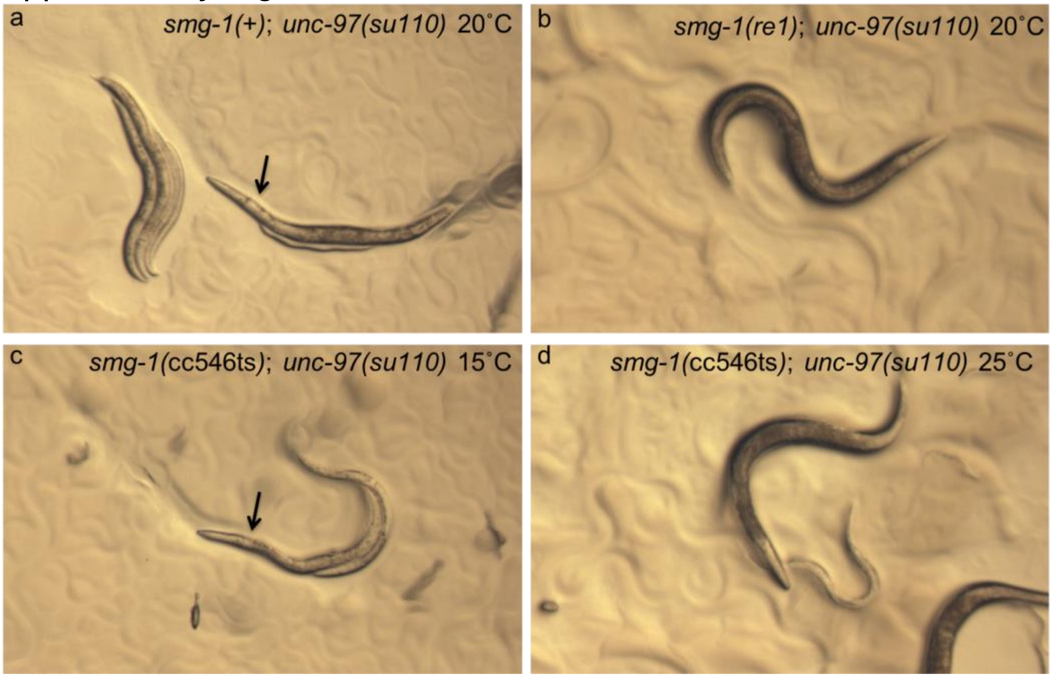

Supplementary Figure 4: TS NMD-sensitive *unc-97(su110)*. Upon crossing into the reference strain for *unc-97(su110)*, HE110, we observed that the strain contained a background mutation partially suppressing the Unc phenotype of *unc-97(su110)*. Whole genome sequencing of this strain identified a nonsense mutation in *smg-1*, which we named *re1* (see **Supplementary Figure 6**).). Photomicrographs were captured from agar plates with 25 msec exposures under same lamp settings. Body posture is representative of locomotion and hence PINCH production by the *unc-97* gene and its NMD-sensitive mutation in the *unc-54* 3'UTR, *r293*: deep body bends represent typical locomotion, shallow bends represent flaccid paralysis. Arrows point to a clear area posterior to the pharynx that indicates a clear patch in the intestine that indicates distension with liquid due to defective defecation. **a)** *unc-97(su110)* animals alone are paralyzed, Egl, and constipated. **b)** These phenotypes are suppressed by the *smg-1(re1)* mutation crossed back into the *unc-97(su110)* background, **c)** not suppressed by *smg-1(cc546*ts*)* at 15˚C but **d)** suppressed by *smg-1(cc546*ts*)* at 25˚C. Mutants for *unc-97* have been reported to have mechanosensory defects (Chen and Chalfie, 2014), and are thereby sluggish and do not move on plate assays. Consequently, we did not include *unc-97(su110)* in our locomotion analysis for **Supplementary Figure 5**.

Pedone Supplementary Fig. 5

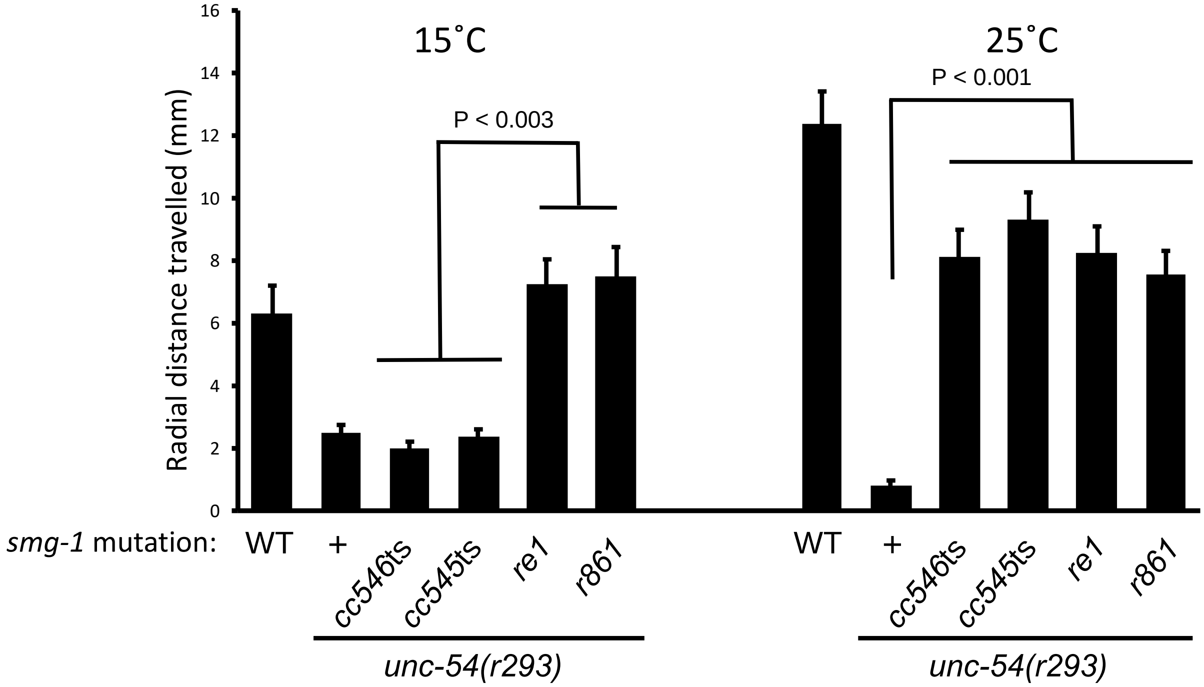

Supplementary Figure 5. Quantification of temperature-dependent locomotion regulated by NMD. Putative null mutations in *smg-1* suppress locomotion defects conferred by the aberrant *unc-54(re293)* 3'UTR at both 15˚C and 25˚C. *smg-1(cc545*ts*)* and *smg-1(cc546*ts*)* fail to rescue locomotion defects at 15˚C but rescue at 25˚C. All animals were scored in sequential assays on the same day, 20 minute assays, then transferred to -20˚C for 5 minutes to arrest locomotion, then counted.

75 Pedone Supplementary Fig. 6

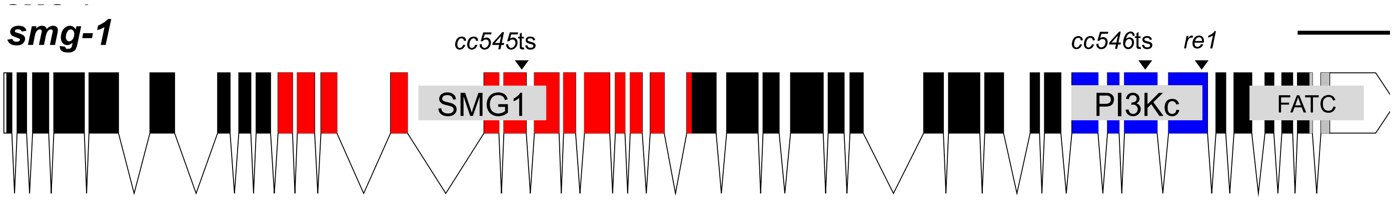

Supplementary Figure 6: Identification of lesions in *smg-1.* The exon-intron boundaries of the *smg-1* gene are shown. Domains are SMG1 (red), PI3Kc (blue) and FATC (gray), UTRs are white. Scale bar = 1000 bp. *cc545*ts is an ACA>ATA transition in exon 15 that causes a T761I missense change. *cc546*ts is an unusual ATG>TTG transversion in exon 35 that causes a M1957L missense change. *re1* is an unusual GAG>TAG transversion in exon 36 that causes an E2093* nonsense change. *cc546*ts is detectable by SNP-snip: restriction enzyme MsI I cuts the wild-type but not the mutant sequence.

Table S1: Lethality conferred by component reagents

Genotype	15˚C	23˚C
*smg-1(cc546*ts); *reIs8[P*$_{lin\text{-}26}$*::GFP::NMD*^{S} *3'UTR]*	1.9% (4/216)	1.4% (6/423)
*smg-1(cc546*ts)	0.9% (3/337)	2.0% (10/522)
reIs8[P$_{lin\text{-}26}$*::GFP::NMD*^{S} *3'UTR]*	1.1% (3/284)	1.2% (5/431)

Pedone Supplementary Figure 7
Domain codes based on SMART
Green: SMG1
Blue: PI3Kc
Gray: FATC
Red: mutated residue

```
CLUSTAL O(1.2.4) multiple sequence alignment

DmnonC        MK-------------------------------NAIHPENCNGAGTE-EEASSAFH---   24
HsSMG1        MSRRAPGSRLSSGGGGGGTKYPRSWNDWQPRTDSASADPDNLKYSSSRDRGGSSSYGLQP   60
CeSMG-1a      ------------------------------------------------------------   0


DmnonC        ---AEIDRVLLNNNGNHGDSSNEGGGGNGSGRGGATGSGNIAGLGGSESMWSPGGGKSHD   81
HsSMG1        SNSAVVSRQRHDDTRVHADIQNDEKGGYS-----------------------VNGGSGEN   97
CeSMG-1a      ------------------------------------------------------------   0


DmnonC        V--AQAFANALLLRNMNHVVGKGQPVVQNHRKAY---QCKGDTINPMANGEDLRLSKIIR   136
HsSMG1        TYGRKSLGQELRVN---NVTSPEFTSVQHGSRALATKDMRKSQERSMSYSDESRLSNLLR   154
CeSMG-1a      ----------------------------------------------MITSRNNDIGNLIE   14
                                                            *  . :  :.:::.

DmnonC        RLINENNPT-VSLELCSKLDQAVRTPINMGYMTCSFVWILDNMLT--LYKQCPPPVLEEC   193
HsSMG1        RITREDDR-DRRLATVKQLKEFIQQPENKLVLVKQLDNILAAVH-DVL-NESSKLLQELR   211
CeSMG-1a      QFRQRDTPQKERKAILARIEEILQTTKNVESLCVKWTYLLDNLCWPSLTKHDRNDMKTLA   74
              :: ..:           ::.: ::   *   :  .   :*  :    * :.    :

DmnonC        SKTLGLIGFINRK---SYPIYEEFIVKNYKSSKRMQKYMIMAL-----------------   233
HsSMG1        QEGACCLGLLCASLSYEAEKIFKWIFSKFSSSAKDEVKLLYLCATYKALETVGEKKA---   268
CeSMG-1a      GKVIRLVGV----LLFDTESY--------------PEFLIYLGTLYQSVTKKSEETRADI   116
               :    :*.       .                     ::

DmnonC        -------RATLSCDTKCEL-HMYADKIMLLLKDFLEN--AESADIFIVVSNTLVQFAASY   283
HsSMG1        -F------------------SSVMQLVMTSLQSILEN--VDTPELLCKCVKCILLVARCY   307
CeSMG-1a      VFSVYFIVGVISQKTENRLIATDTENVEKSLDWIIKVLPNSSISVYNHCLKGFVLVANTF   176
                                      : :   *. :::    .: .:     : :: .*  :

DmnonC        AETFECHFTDVVDIVIGWQLEAGQPTDLKTHCAQVLEQLTPFFSKQIDFSYGLLDQFVED   343
HsSMG1        PHIFSTNFRDTVDILVGWHIDHTQKPSLTQQVSGWLQSLEPFWVADLAFSTTLLGQFLED   367
CeSMG-1a      PNVYAAMFESTLRAILTNLPDFNS------HEKNF----ELLIDTVMRFSDQ-----LNE   221
               . :   * ..:  ::    :  .      :          :    : **       :::

DmnonC        ITTLEEGE---------------PANTAERVGAFVGAFNTLLKCLA-RMQIFVGMPTCEC   387
HsSMG1        MEAYAEDLSHVASGESVDEDVPPPSVSLPKLAALLRVFSTVVRSIGERFSPIRGPPITEA   427
CeSMG-1a      KPHLAEEMVRII----------RPDIKKNGLGNM--------RELKKRM------KLTMA   257
                   *                 *  .   :. :        : :  *:          .

DmnonC        IVKMAVDHLIKIMPTLHL--NTEALVNI-N--------------ELICICLLN-NFTGLD   429
HsSMG1        YVTDVLYRVMRCVTAANQVFFSEAVLTAANECVGVLLGSLDPSMTIHCDMVIT---YGLD   484
CeSMG-1a      LVKM--AKSQKMLEETNQMI-SEMSIEL-EENGGK-W-SSASLITIVCDVFNELLILGKD   311
               *.    :  : :   :    :*  :   :               : *  .      * *

DmnonC        PILLEQVLLDQVK------RMISLTELQRQSVLYLLLCTVRRLRARLT-PSLVHFIFQSN   482
```

```
HsSMG1          QLENC-----QTCGTDY-----------IISVLNLLTLIVEQINTKLP-SSFVEKLFIPS  527
CeSMG-1a        DVKLQKGVEESLCNVLKDLNLSNQSTMEKQAFFNSLAKIVKQLPAESQVKTRVHQIVFNT  371
                 :        .                   :.:  *   *.:: :.    : *. :.  .

DmnonC          ------PYMTKVRLRSPGETSYKLLLRTCQETLLIRNVPLLQQAYKYLVDDIDACLEKLL  536
HsSMG1          SKLLFLRYHKEKEVVAVAHAVYQAVLSLKNIPV-------LETAYKLILGEMTCALNNLL  580
CeSMG-1a        ETGLFTPKNRDNRM-FGHNMIYKDLINLVSVLLTPTSLNHLQATYTDLRKIMIDS-----  425
                          . .:    .  *: ::   .  :       *: :*. :   :  .

DmnonC          IT----------------APRSKARKASVLLVFHLSALAALAKQTSSIIGMYACKPSILE  580
HsSMG1          HSLQLPEACSEIKHEAFKNHVFNVDNAKFVVIFDLSALTTIGNAKNSLIGMWALSPTVFA  640
CeSMG-1a        --------MSRLKQSEDTPYSDNIRWNESILLLFFSSLQSISCAKSSLIVMMGIRPSIFE  477
                      :    . :::: :*:* ::.  ..*:* * .  *:::

DmnonC          LLLTNCRAHELKFWSKYPAAQQAIFGLLVVHCQANHNFRTNSSL-----LRDQELSAENT  635
HsSMG1          LLSKNLMIVHSDLAVHFPAIQYAVLYTLYSHCTRHDHFISSSLSSSSPSLFDGAVISTVT  700
CeSMG-1a        FFSSELPLTEYWLASNHPEVYHLFITIFVGHLKAHDFYIVQSD-----YIVRGDSIGQSI  532
                :: .:    .  :  :.*     .:  :  *   :. :  .*       :      .

DmnonC          SPTANSFA--------SILRFLDSVLGQAHQLAPQNLRVLLQWIQMLLRE-CRE-KIDLL  685
HsSMG1          TATKKHFS--------IILNLLGILL-KKDNLNQDTRKLLMTWALEAAVLMKKSETYAPL  751
CeSMG-1a        GQTKRDYARKQVVALQKIINNFG------DKLWKKTRLMISSWLHSLIATACEHQIGSDS  586
                  * . ::         *:. :.      .:*  ..  ::  *         .

DmnonC          MEQENFRGICRNIAATASKLVPLESAACIQTVLDYGLERLEKYPKLLILYRD--------  737
HsSMG1          FSLPSFHKFCKGLLANTL---VEDVNICLQACSSL--------HALSSSLPDDLLQRCVD  800
CeSMG-1a        FSQREWVRLRNTVIHQSV---LTWNNECVNQALTIL-STATKWSELTSDIHRDIADKTKK  642
                :.  .:  : . :   :          *::               *

DmnonC          ------TALQQLQMLSTNYHAPYFQIYAQLPLHLTLTGGESSMPG---MASRRV----SV  784
HsSMG1          VC---RVQLVHS---GTRIRQAFGKLLKSIPLDVVLSNNNHTEIQEISLALRSHMSKAPS  854
CeSMG-1a        AKWKEATTIWESGDCNTYIRQSMSTVY--------------------QMSQERQQKTITS  682
                      . : .    .*  :     :                      :: .

DmnonC          WQQRISQYSAVRDNVFRDFFDRVQKPEQDSLIHCLRELFVRSCQVAPQDERQMNLSQCTK  844
HsSMG1          NTFHPQDFSDVISFILYG---NSHRTGKDNWLERLF----YSCQRLDKRDQS-TIPRNLL  906
CeSMG-1a        TSFGAEEFIIITNFLLKQATPTTFKKGQNSWMDEVLETFTQGCRTLEKPDS--YVPE---  737
                     .::  : . ::        :  ::. :. :      .*:   : :    : .

DmnonC          RCQRLAIAWLQFEAARYCVDQRLRTTVGKPQETFLGFEAIIMRHARLLSGCAKE---IER  901
HsSMG1          KTDAVLWQWAIWEAAQFTVLSKLRTPLGRAQDTFQTIEGIIRSLAAHTLNPDQDVSQWTT  966
CeSMG-1a        -TFIEKWDWIINQTANFCIVNKMKTPLGKPMQTFAAFENEIKRLAKEVIVRKNSDKKLNK  796
                        *   ::*.: : .:::* :*:  :**  :*  *   *       :.

DmnonC          SALDDLSLE---ELLSMQSNLSLLLGFLDALEKLIYNAAEGSA--FALRPPEKQVAAFFR  956
HsSMG1          ADNDE-------GHGNNQLRLVLLLQYLENLEKLMYNAYEGCAN--ALTSPPKVIRTFFY  1017
CeSMG-1a        SSTEDPNQSPPLKYSVQWLRVHLLLKLIVVLEKLMNSAIHGGSSVFNLTEIPVSSRQFFT  856
                :  ::              .: ***  :  ****: .* .* :    *         **

DmnonC          LNNPTCQSWFNRIRIGVVIIAMHVQQPELVIRYAQQILVNSKTQDPTYSQAI--------  1008
HsSMG1          TNRQTCQDWLTRIRLSIMRVGLLAGQPAVTVRHGFDLLTEMKTTSLSQGNELEVTIMMVV  1077
CeSMG-1a        VNAASCEVWLNRVYYPALLVAYFNGYYGLVIRFGSNALSHFARQKDGDNDK------KIV  910
                 *  :*: *:.*:    : :.       :.:*.. : * .    .   .:

DmnonC          ------VYMAWSLVSCQEADSLRGLRLWARGKSCKSY--KWLKYAADQAAGKRESALAGY  1060
HsSMG1          EALCEL----------HCPEAIQGIAVWSSSIVGKNL--LWINSVAQQAEGRFEKASVEY  1125
CeSMG-1a        NGVCTASLMSLSMAVLGEPMEIVGLRRKVREEFGTDMGQSLMEALGEMANARYETALVAL  970
```

```
                                   : *:         ..     ::  .: * .: *.* .



DmnonC        RTILAEKELQSELE----------------------------------------PHTRQF   1080
HsSMG1        QEHLCAMTGVDCCISSFDKSVLTLANAGRNSASPKHSLNGESRKTVLSKPTDSSPEVINY   1185
CeSMG-1a      EAVLVTDAATN----ETLKMIIQLAM----------------------------VDILNR   998
              .  *      .                                            .  :

DmnonC        VVSQMMQCLQDLGQWSQLVELKQQQMTRPEDRELNPFLQRSNVEVNALERLLAKSEESCS   1140
HsSMG1        LGNKACECYISIADWAAVQEWQNAIHDLK-------------------------------   1214
CeSMG-1a      IRLPQATDYYKV------VLFGE-------------------------------------   1015
              :         .:          :

DmnonC        SMDALGGVFQQLSLWPSNWDESVSSSGLSERASFSSIHMRQRTE----------------   1184
HsSMG1        --------------------KSTSSTSLNLKADFNYIKSLSSFESGKFVECTEQLELLPG   1254
CeSMG-1a      -----------------------ESNDSTITEDFRSVELLTKFEKL--------------   1038
                                     .*.. .   .*  :.     *

DmnonC        ------------DIVLHKLLED---------------------RCVPDQAKN-LLDTQWR   1210
HsSMG1        ENINLLAGGSKEKIDMKKLLPNMLSPDPRELQKSIEVQLLRSSVCLATALNPIEQDQKWQ   1314
CeSMG-1a      ----------------------------------------------NNTVNEKRQVVDWS   1052
                                                                :      .*

DmnonC        DSLL----NPSFDQRSCKELTLLRH-IVQGVSGGQELSLLPVSSG-----RCQNR-----   1255
HsSMG1        SIT------ENVVKYL-KQ-------TSRIAIGPLRLSTLTVSQSLPVLSTLQLYCSSAL   1360
CeSMG-1a      ARERFQFVESAFSQTM-RRTELLDIQKDFSAMGALALSADS---------SCKLYSDI--   1100
                         . :   :.           . *   **              :

DmnonC        ------------------SKFISSAILMRCLAWTQLLRQHCAPGS---------------   1282
HsSMG1        ENTVSNRLSTEDCLIPLFSEALRSCKQHDVRPWMQALRYTMYQNQLLEKIKEQTVPIRSH   1420
CeSMG-1a      --------SSTSLIIANL-----VNKMTGVSQWKNKLTDTEIFDRNEEGNDGDKLAICRK   1147
                                              * : *      .

DmnonC        WETLCLDAAAAAREEGNLQLAETLLTQFFGQPIGEIAAL-----FSLEQGVQTDNPEMLR   1337
HsSMG1        LMELGLTAAKFARKRGNVSLATRLLAQCSEVQLGKTTTAQDLVQHFKKLSTQGQVDEKWG   1480
CeSMG-1a      LMHWGRHTK---SNRGQSCAAH-----SEIIRLSRKTSNCELAFFHINSAIRGEKLAAWQ   1199
                     :     :.*:   *           :.. ::      .  : . : :

DmnonC        GYSEL--VKCLHLQQQQSQTHSGDLSSSIDVCAAL-CLNIQKSNNQPAAGADLLLNLADW   1394
HsSMG1        PELDIEKTKL--LYTAGQSTHA---MEMLSSCAISFCK----SVKAEYAVAKSILTLAKW   1531
CeSMG-1a      R-LEVERQRLKLVKTQNLDVRIREMNEVFGSLAEVFTTSC--QLKSDFQMVDGMIK-EKM   1255
                 ::   :   :     ..:     . :.  *         . :     .. ::.  .

DmnonC        IAVRTCNGL-----------------------------TTNQSPVLIQLLDQLPECPLTC   1425
HsSMG1        IQAEWKE-ISGQLKQVYRA--QHQQNFTGLSTLSKNILTLIELPSVNTMEEEYPR--IES   1586
CeSMG-1a      ISEGYNEDIAKREEHMSRASIQLADFFQSLPELE-NVLAPNLFPTII-----WSE--LQR   1307
              *     : :                             :    * :        .  :

DmnonC        DSSQPLAIPQAERMVARLVHSCLQQRPNYAEALIAYGNWCYRWGKKVADSCCVLTQADAT   1485
HsSMG1        ESTVHIGVGEPDFILGQLYHLSSVQAPEVAKSWAALASWAYRWGRKVVDNASQGEGVRLL   1646
CeSMG-1a      R-SDSL-SAGYHGIVGSLFHLASEMCPSLAKAHLKMARWAYEIAKIKNF-----------   1354
                :  :     . ::. * * .    *. *::    . *.*. .:

DmnonC        ---AISQALDIPQPLESEKLDELLQALS---TEQPPANCV-EVCPDAARARDDE------   1532
HsSMG1        PREKSEVQNLLPDTITEEEKERIYGILG-QAVCRPAGIQDEDITLQITESEDNEEDDMVD   1705
CeSMG-1a      PAENLSFYKFGKDE---TENEELLKSLEATSLVNLEKL------VRAAISDDLRA-----   1400
```

```
                     .      :     : :.:   *      .             : : * .

DmnonC          -AAKNRLRRLTFLADKTPEALDAILQIWRRAIANTYDYYKDAARSYFQYLSFKSGSGPEK  1591
HsSMG1          VIWRQLISSCPWLSELDESATEGVIKVWRKVVDRIFSLYKLSCSAYFTFLKLNAGQIPLD  1765
CeSMG-1a        --------------NNILAPNSHFMHIWKMVRDHRTKFLSIAVTSYFQFIQNMSGDCD--  1444
                              :      . .:::*: .  .  .  . :  :** ::.  :*.

DmnonC          PEGEGVVSQRERLHVDDSNLVTTTLRLLRLIVKHASGLQEVLEQGLHTTPIAPWKVIIPQ  1651
HsSMG1          EDDPRLHLSHRVEQSTDDMIVMATLRLLRLLVKHAGELRQYLEHGLETTPTAPWRGIIPQ  1825
CeSMG-1a        ----------NLPYSKKEETTLATLRILELLVKHGDVLIDVINDGLNKTNVHIWKEILPQ  1494
                          .     ..  . :***:*.*:***.. * : ::.**..*    *: *:**

DmnonC          LFSRLNHHEPYVRKSVCDLLCRLAKSRPQLVIFPAVVGANREQQDATA------------  1699
HsSMG1          LFSRLNHPEVYVRQSICNLLCRVAQDSPHLILYPAIVGTISLSSESQASGNKFSTAIPTL  1885
CeSMG-1a        LFARLSHPSEHIRKTLVDLISKICTAAPHAVVFQVVSGAASSSTDG--------------  1540
                **:**.* . ::*::: :*:.::.   *: ::: .: *:   . :.

DmnonC          ------------------PPATA------------RPTTEDACCYGYLLGELSKQAPEAV  1729
HsSMG1          LGNIQGEELLVSECEGGSPPASQDSNKDEPKSGLNEDQAMMQDCYSKIVDKLSSANPTMV  1945
CeSMG-1a        ---------------------------EELEEQQNDDRNRVRACCEKLETNMSQSYPNLV  1573
                                                           *   :  ::*.  *  *

DmnonC          QHVKLMVKELRRVCLLWDEYWIHSLAHIYNTYVSRVSALATDFRPDDH---EGKNNRF--  1784
HsSMG1          LQVQMLVAELRRVTVLWDELWLGVLLQQHMYVLRRIQQLEDEVKRVQNNNTLRKEEKIAI  2005
CeSMG-1a        KDVRQFVAELERINLLNEEKWSVVMGTMEHEMEKRLSLIRTENAKTESALHLTASVKNDI  1633
                 .*: :* **.*: :* :* *   :         *:. :  :    :       . :

DmnonC          -----NVWRPQLLADLEA-LVAVTSRPPETTYERSFRKRFDAPIRLTVDALR----HRRY  1834
HsSMG1          MREKHTALMKPIVFALEHV-RSITAAPAETPHEKWFQDNYGDAIENALEKLKTPLNPAKP  2064
CeSMG-1a        IVKRTQLLTRQIFDVLDELYQQTVIEPPKSKNEEEFVTAFAEVLTNAFQESRI-SRTTSP  1692
                           :.  *:      .  * ::  *. *   :   :  :.:  :

DmnonC          PEAWDKLKQLYHILQSNMIRGSGSTLKMQSISPVLCGIGRMRISMPGLDAHGPDGDQVYI  1894
HsSMG1          GSSWIPFKEIMLSLQQRAQKRASYILRLEEISPWLAAMTNTEIALPGEVSA---RDTVTI  2121
CeSMG-1a        EKSWIPFKNLIANFVHRNSKKGMQTFETEDISPYLASLSNSCVPMPGQESVE-FDRVVSI  1751
                 .:*  :*::   :  .  : .   :. :.*** *..: .  : :**  :       * *

DmnonC          ESVESSVCVLPTKTKPKKVAFYGSNGQRYTFLFKGMEDLHLDERIMQFLSISNAIMACRS  1954
HsSMG1          HSVGGTITILPTKTKPKKLLFLGSDGKSYPYLFKGLEDLHLDERIMQFLSIVNTMFATI-  2180
CeSMG-1a        SRVARQVTILPTKTRPKKLGFVGSDGKQVAFLFKGREDLHLDERVMQFLRLCNVMLQPGK  1811
                  *   : :*****:***: * **:*:   :**** ********:**** : *.::

DmnonC          --DAPGNGCYRAHHYSVIPLGPQSGLISWVDGVTPVFALYKKWQQRRSQVAGNAGAGA--  2010
HsSMG1          --NRQETPRFHARHYSVTPLGTRSGLIQWVDGATPLFGLYKRWQQREAALQAQKAQDSYQ  2238
CeSMG-1a        GKHRQSVAAYQAHHYAVIPLGPRSGLIKWVEGATPMFHIYRKWQMKEKALKQATKKNGET  1871
                  .      ::*:**:* *** :****.**:*.**:* :*::** :.  :      ..

DmnonC          ---VANVPRRFTDLFYNKLSPLLAKHNM--QVSDPRRQWPISVLLQVLDELSQETPNDLL  2065
HsSMG1          TPQNPGIVPRPSELYYSKIGPAL--KTVGLSLDVSRRDWPLHVMKAVLEELMEATPPNLL  2296
CeSMG-1a        VP----EIERPSNMYHNMIRLAFADHKIDSSITSDRSKWPAEILEEVFESLTAKTPTDLI  1927
                         * :::::. :   :  :.:  .:   * .**  ::  *::.*   ** :*:

DmnonC          ARELWCQAGNAAEWRQSVRRFVRCMSVMSMIGYVIGLGDRHLDNVLINLGSGDIVHIDYN  2125
HsSMG1          AKELWSSCTTPDEWWRVTQSYARSTAVMSMVGYIIGLGDRHLDNVLIDMTTGEVVHIDYN  2356
```

```
CeSMG-1a      SRELWMRANDATTWWSVTKRYSRSLAVMSMVGSVLGLGDRHLDNLLVDLKWGHVVHIDYN   1987
              ::***  .     *   .: :  *. :****:* ::*********:*:::  *.:******




DmnonC        VCFEKGRTLRIPEKVPFRLTQNLVQAMGITGIEGPFRLGCEYVLKVMRKERETLLTLLEA   2185
HsSMG1        VCFEKGKSLRVPEKVPFRMTQNIETALGVTGVEGVFRLSCEQVLHIMRRGRETLLTLLEA   2416
CeSMG-1a      ICFDKGKNLRIPETVPFRLTRNMRHALGPSEMYGTFRESCVHVLSTLRSGHQVLTMLLDA   2047
              :**:**:.**:**.****:*:*:  *:* : : * ** .*  **  :*  ::.*  **:*

DmnonC        FVYDPLVDWTTNDDAQALRRSLNAKLQESADGGGAGGLGVGDLKYHKKDKNKGKPLDSDV   2245
HsSMG1        FVYDPLVDWTAGGEAGFAG-------AV--YGGGGQ---------QAESKQSKREMEREI   2458
CeSMG-1a      FVFDPLVDWTSHEHTATSGVSLALQLAV--YGSNWK---------TKAKER------LTD   2090
              **:*******:  .:                *..               .:.

DmnonC        KRQPFLSKLGMLQKYWSTNKTELMPQLEEMEQEVGNLQAAQAKQVVA---------EEEL   2296
HsSMG1        TRSLFSSRVAEIKVNWFKNRDEMLVVLPKLDGSLDEYLSLQEQLTDVEKLQGKLLEEIEF   2518
CeSMG-1a      AMELLNLRMSEVQTLWLANRDDLLHWMKQVTECLL----IENSMLGANAIY----AQQRV   2142
                . :  ::. ::  *  *: :::  : ::   :      : .   .         : ..

DmnonC        V---------------KLNQRSALIAEIKSLGTAIE--------SHSFNTASLRNAVRRG   2333
HsSMG1        LEGAEGVDHPSHTLQHRYSEHTQLQTQQRAVQEAIQVKLNEFEQWITHYQAAFNN-----   2573
CeSMG-1a      KAG--------TELREAVTRHHALAKELRPLIRVIGKEREEFADYLKFYKQALFDPLLKG   2194
                                ..:  *  : : :  .*           ..   :: :

DmnonC        HSEALALLSTERLPDFGRVQCILRSYGQCLQLYHLLDLQGQLVKLQMESNSENAR-----   2388
HsSMG1        --------------------------------------------LEATQLASLLQEISTQ   2589
CeSMG-1a      HSALRNELDI--------DTCVY---NFNIVMQNIDNVFGALVNLSFTPIETITSR-TSQ   2242
                                                          *.

DmnonC        -EFS-----ALTEALQLSGLDSMRSQLNELLGRMDMVAQKSS--------------KHLQ   2428
HsSMG1        MDLGPPSYVPATAFLQNAGQAHLISQCEQLEGEVGALLQQRRSVLRGCLEQLH-------   2642
CeSMG-1a      QEFKPPPGLENVWVVK---------QDQQENSQAREVVRRVERRLNGWLDG---------   2293
               ::        .  ::         * ::  ..   : ::

DmnonC        EYAGV---MNFYPE---QSHRQNLFVRFHDSFATY-----IQNGYTADSTTNTNSPSSSI   2477
HsSMG1        HYATV---ALQYPKAIFQKHRIEQWKTWMEELICNTTVERCQELYRKYEMQYAPQP-PPT   2698
CeSMG-1a      ------------------------------------------------------------   2322
                  .        .    .:  : :  :

DmnonC        ICKADVVGVAEAMEYSWERLGCQL----------------------------HEASKLYA   2509
HsSMG1        VCQFIT-----ATEMTLQRYAADINSRLIRQVERLKQEAVTVPVCEDQLKEIERCIKVFL   2753
CeSMG-1a      ------------------------------------------------------------   2322


DmnonC        ANQAQALTLGAPTTALLSMIVQSGCSQLLLKASLVRTLDRAGGAFAAYEQ-VALASHDDG   2568
HsSMG1        HENGEEG-----SLSLASVIISALCTLTR------RNLMMEGAASSAGEQLVDLTSRDGA   2802
CeSMG-1a      ------------------------------------------------------------   2322


DmnonC        LL--------------------------------------------------------HH   2572
HsSMG1        WFLEELCSMSGNVTCLVQLLKQCHLVPQDLDIPNPMEASETVHLANGVYTSLQELNSNFR   2862
CeSMG-1a      ------------------------------------------------------------   2322


DmnonC        QLLFIHLVRTMLQGVLVMTKE---EDQHLA------QLESLLSALSHLKK----MFEYDL   2619
```

```
HsSMG1          QIIFPEALRCLMKGEYTLESMLHELDGLIEQTTDGVPLQTLVESLQAYLRNAAMGLEEET  2922
CeSMG-1a        ------------------------------------------------------------  2322

DmnonC          PAN---LYR------LLLLQPNLGKLSALCHLSASSLAQLFLEATMENGHKPPDQFPVER  2670
HsSMG1          HAHYIDVARLLHAQYGELIQPRNGSVDETPKMSAG------------------------Q  2958
CeSMG-1a        ------------------------------------------------------------  2322

DmnonC          RFLLTLQPVYDQFLLASTSLDSLVSSMQSMLEDV-----HDVQTQQIMELGLM-RSCHTE  2724
HsSMG1          MLLVAFDGMFAQV---ETAFSLLVEKLNKMEIPIAWRKIDIIREARSTQVNFFDDDNHRQ  3015
CeSMG-1a        ------------------------------------------------------------  2322

DmnonC          LNDECFF-----------G---LVSEALESS------------------------RTCDV  2746
HsSMG1          VLEEIFFLKRLQTIKEFFRLCGTFSKTLSGSSSLEDQNTVNGPVQIVNVKTLFRNSCFSE  3075
CeSMG-1a        ------------------------------------------------------------  2322

DmnonC          REMARPMLGFIHRLQVEKLAGL----LPILTRNFYTAVGPQCLPTASCG-----DPAQAD  2797
HsSMG1          DQMAKPIKAFTADFVRQLLIGLPNQALGLTLCSFISALGVDIIAQVEAKDFGAESKVSVD  3135
CeSMG-1a        ------------------------------------------------------------  2322

DmnonC          HLCESLFISLQSDGALLQ---QQAEI--------------------ALLSQQVDLH--TL  2832
HsSMG1          DLCKKAVEHNIQIGKFSQLVMNRATVLASSYDTAWKKHDLVRRLETSISSCKTSLQRVQL  3195
CeSMG-1a        ------------------------------------------------------------  2322

DmnonC          AASAQYWAYSEALGSQLRCGPHIVSRPKLTAAIGECWLELDQKLTALQQLQAGLES---Q  2889
HsSMG1          HIAMFQWQHEDLLINRPQ--AMSVTPPPRSA----ILTSMKKKLHTLSQIETSIATVQEK  3249
CeSMG-1a        ------------------------------------------------------------  2322

DmnonC          ---LSQLQTQRSNWNRNHIDNLLRMEQCNKQRTMSHVALLQKMTDGAGAVARLEQNAIV-  2945
HsSMG1          LAALESSIEQRLKWAGGANPALAPVLQDFEATIAERRNLVLKESQRASQVTFLCSNIIHF  3309
CeSMG-1a        ------------------------------------------------------------  2322

DmnonC          ------------VGEEGQALVDHLEQWLAAHGQWQASSSRIS--AVEQSMVELLDPEGAI  2991
HsSMG1          ESLRTRTAEALNLDAALFELIKRCQQMCSFASQFNSSVSELELRLLQRVDTGLEHPIGS-  3368
CeSMG-1a        ------------------------------------------------------------  2322

DmnonC          DHYWLENVQGLLEEQTCKVHREIAAIEGEQQSKHRFICTLLKETLR--------------  3037
HsSMG1          -SEWLLSAHKQLTQDMS-T---QRAIQTEKEQQIETVCETIQNLVDNIKTVLTGHNRQLG  3423
CeSMG-1a        ------------------------------------------------------------  2322

DmnonC          ----LLENMPRFHVQSLC------------------------------SEAQAQGQGKM-  3062
HsSMG1          DVKHLLKAMAKDEEAALADGEDVPYENSVRQFLGEYKSWQDNIQTVLFTLVQAMGQVRSQ  3483
CeSMG-1a        ------------------------------------------------------------  2322

DmnonC          -EYANVQLLSDHLREGQGLMQSLYMRLQELRKDI-------CSDR---RVLQPSMLQNWR  3111
HsSMG1          EHVEMLQEITPTLKELKTQSQSIYNNLVSFASPLVTDATNECSSPTSSATYQPSFAAAVR  3543
CeSMG-1a        ------------------------------------------------------------  2322
```

```
DmnonC         HQL------EMILTLAKQEVNEFFKGLE----DFMQHAGETDSYE-IFTHAKGSGNVHEQ  3160
HsSMG1         SNTGQKTQPDVMSQNARKLIQKNLATSADTPPSTVPGTGKSVACSPKKAVRDPKTGKAVQ  3603
CeSMG-1a       ------------------------------------------------------------  2322


DmnonC         KRNAYGVSVWKKIRMKLEGRDPDSNQRSTVAEQVDYVIREACNPENLAVLYEGWTPWV    3218
HsSMG1         ERNSYAVSVWKRVKAKLEGRDVDPNRRMSVAEQVDYVIKEATNLDNLAQLYEGWTAWV    3661
CeSMG-1a       --------------------SAGPDRKLSPREEADILIAEATSTPNLSQMYEGWTAWV    2322
                                   .:  .:: :: *.:*::* **::  **  :********
```

Supplementary Figure 7: Alignment of SMG-1 orthologs from different species. Shown is a Clustal Omega alignment on default settings, with a single manual curation to align the C-terminal FATC domain, where the *C. elegans* sequence was not aligned, likely due to gap penalties. Sequences are *Drosophila melanogaster* nonC (DmnonC), *Homo sapiens* SMG1 (HsSMG1 and *Caenorhabditis elegans* SMG-1a (CeSMG-1a). Domains predicted by SMART are highlighted with a key at the beginning. The *cc545*ts and *cc546*ts mutations alter conserved residues in the SMG1 (T761I) and PI3Kc lipid kinase (M1957L) domains, respectively (bold, underlined, red).

Pedone Supplementary Fig. 8

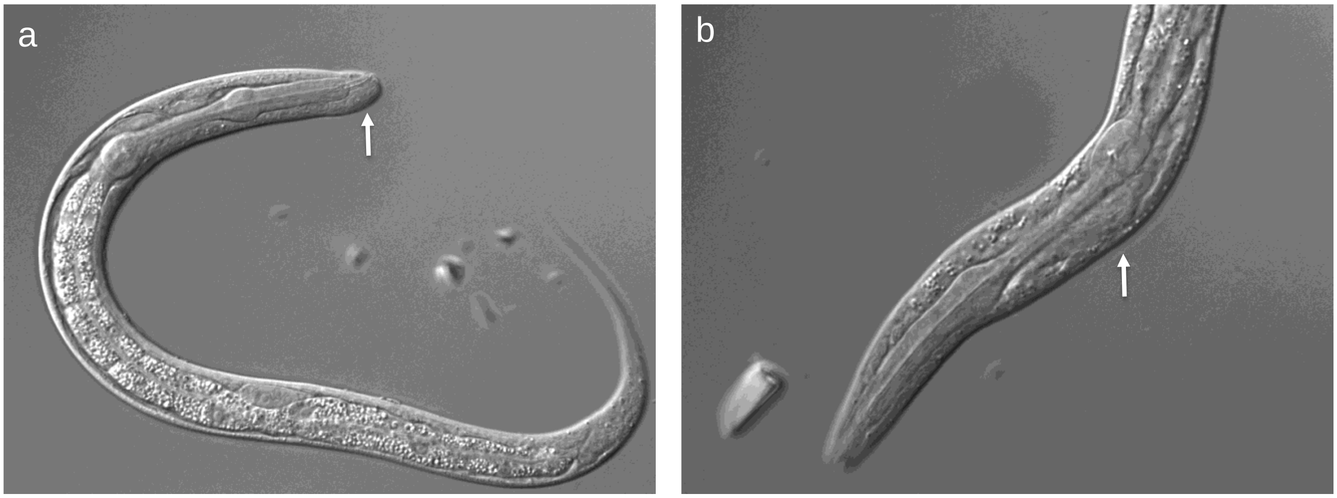

Supplementary Figure 8. Weak morphogenetic phenotypes. Low penetrance and low expressivity phenotypes are caused by the GFP over-expressing strain *smg-1(cc546*ts*)*; *reIs8[Plin-26::gfp::NMD*^{S}*3'UTR]*, perhaps due to over-represented promoter sequences titrating factors important for morphogenesis. Arrows indicate mild bulges in the animal's epithelium, frequently around the head in animals grown at 23˚C. Occasional animals with such bulges grew slower, presumably due to compromised feeding. In this experiment GFP lethality = 0.8% (4/453), WT lethality = 0.6% (4/671).

Supplementary Table S2: EGL-1/BH3-only-induced lethality is caspase-dependent

Genotype	RNAi	% lethality[a]
*smg-1(cc546*ts*)*; *reIs14[*$P_{lin\text{-}26}$*::egl-1(+)::*NMD^{S} *3'UTR]*	*Luc.*	98.5 (321/326)
*smg-1(cc546*ts*)*; *reIs14[*$P_{lin\text{-}26}$*::egl-1(+)::*NMD^{S} *3'UTR]*	*ced-3*	84.8 (245/289)
*smg-1(cc546*ts*)*; *ced-3(n717)*; *reIs14[*$P_{lin\text{-}26}$*::egl-1(+)::*NMD^{S} *3'UTR]*	*Luc.*	11.4 (22/193)

[a]Animals were grown at 25˚C.

Pedone Supplementary Fig. 9

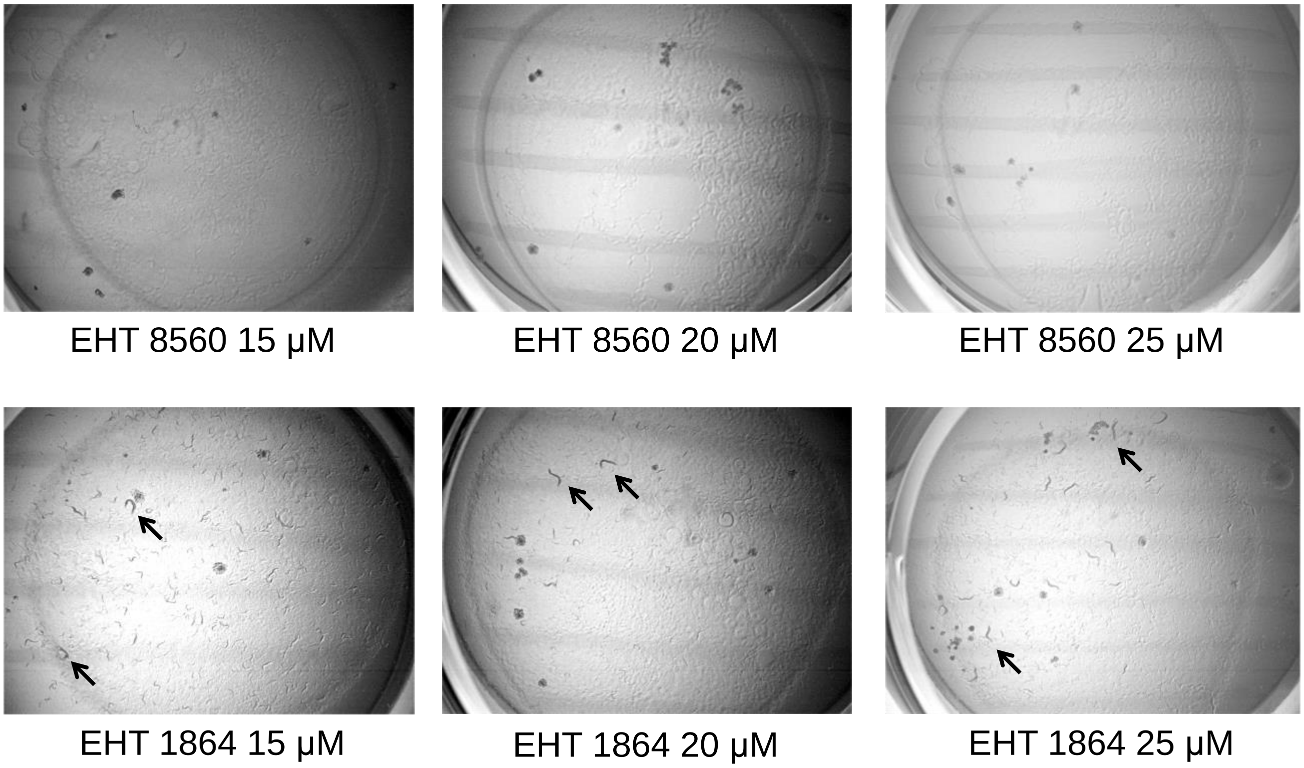

Supplementary Figure 9. EHT 1864 rescue vs. negative control EHT 8560. 6-well plate assays for small molecule rescue of lethality conferred by *smg-1(cc546*ts); *reIs6* lethality at 23˚C. **Top row:** Synchronized groups of animals treated with increasing doses of negative control molecule EHT 8560. None survived to hatching. (Tracks are left by Rol parents that laid the eggs). **Bottom row:** Synchronized groups of animals treated with increasing doses of Rac inhibitor EHT 1864. Arrows indicate a subset of grown adults, but many smaller larvae are evident. In all images, dark spots are salt crystals in the agar. Images were sharpened in Powerpoint to improve visibility.

Supplementary Table 3: *C. elegans* strains used in this study

Strain #	Genotype
DV2135	*reIs6* [P_{lin-26}::*ced-10*(gf)::NMD^S *3'UTR*+P_{myo-2}::*gfp*+*rol-6*(d)] III
DV2149	*smg-1(cc546*ts) I; *reIs6* [P_{lin-26}::*ced-10*(gf)::NMD^S *3'UTR*+P_{myo-2}::*gfp*+*rol-6*(d)] III
DV2271	smg-1(cc546ts) I; max-2(nv162) II; *reIs6* [P_{lin-26}::*ced-10*(gf)::NMD^S *3'UTR*+P_{myo-2}::*gfp*+*rol-6*(d)] III
DV2272	*smg-1(cc546*ts) I; *max-2(nv162)* II; *reIs6* [P_{lin-26}::*ced-10*(gf)::NMD^S *3'UTR*+P_{myo-2}::*gfp*+*rol-6*(d)] III
DV2286	*smg-1(cc546*ts) I; *reIs6* [P_{lin-26}::*ced-10*(gf)::NMD^S *3'UTR*+P_{myo-2}::*gfp*+*rol-6*(d)] III; *unc115(ky275)* X
DV2314	*smg-1(cc546*ts) *pes-7(gk123)* I; *reIs6* [P_{lin-26}::*ced-10*(gf)::NMD^S *3'UTR*+P_{myo-2}::*gfp*+*rol-6*(d)] III
DV2316	*smg-1(cc546*ts) I; *pkn-1(ok1673)* X; *reIs6* [P_{lin-26}::*ced-10*(gf)::NMD^S *3'UTR*+P_{myo-2}::*gfp*+*rol-6*(d)] III
DV2157	*smg-1(cc546*ts) *unc-54(r293)* I
DV2196	*smg-1(re1) unc-54(r293)* I
PD8117	*smg-1(cc545*ts) *unc-54(r293)* I
DV2652	*smg-1(r861) unc-54(r293)* I
DV2208	*unc-97(su110)* X
DV2653	*smg-1(r861)* I; *unc-97(su110)* X
DV2654	*smg-1(cc545*ts) I; *unc-97(su110)* X
DV2658	*smg-1(cc546*ts) I; *unc-97(su110)* X
HE110	*smg-1(re1)* I; *unc-97(su110)* X
DV2208	*unc-97(su110)* X 4x outcrossed
DV2471	*smg-1(cc545*ts) I 2x outcrossed to DV2453
DV2376	*reIs14 [*P_{lin-26}*::egl-1::*NMD^S *3'UTR + rol-6*(d) + P_{myo-2}*::gfp]* 4x outcrossed
DV2437	*smg-1(cc546*ts) I; *reIs14[*P_{lin-26}*::egl-1::*NMD^S *3'UTR + rol-6*(d) + P_{myo-2}*::gfp]*
DV2348	*smg-1(cc546*ts) I; *reIs8 [*P_{lin-26}*::gfp::*NMD^S *3'UTR + rol-6*(d)*]* 4x backcrossed to *smg-1(cc546*ts*)*
DV2453	unc-87(e1459) I 2x outcrosssed
PD8120	*smg-1(cc546*ts) I
PD8119	*smg-1(cc545*ts) I
DV2683	*smg-1(cc546ts)* I*; ced-3(n717)* IV*; reIs14[*P_{lin-26}*::egl-1(+)::*NMD^S *3'UTR]*

Supplementary Table 4: Plasmids used in this study

Plasmid name	Description
pML433	*lin-26(eFGHi) enhancers::minimal myo-2 promoter::GFP*
pPD118.44	*Synthetic NMD*^{S} *3'UTR* (from inverted let-858 coding sequence)
pCM1.3	$P_{lin\text{-}26}$*::synthetic let-858 NMD*^{S} *3'UTR*
pCM1.4	$P_{lin\text{-}26}$*::gfp::synthetic let-858 NMD*^{S} *3'UTR*
pCM3.2	$P_{lin\text{-}26}$*::ced-10(+)::synthetic let-858 NMD*^{S} *3'UTR*
pCM3.3	$P_{lin\text{-}26}$*::ced-10*(gf)*::synthetic let-858 NMD*^{S} *3'UTR*
pCM12.2	$P_{lin\text{-}26}$*::egl-1::synthetic let-858 NMD*^{S} *3'UTR*

Supplementary Table 5: Oligonucleotides used in this study.

Oligos	Sequence	Use
DJR511	TTTTTTggatccttaattaGTggccggccTTTTTCTGAGCTCGGTACCCTCC	P_{lin-26}
DJR459	TTTTTTggatccTggccggccACTCATTTTTTCTGAGCTCGGTACCCTCC	P_{lin-26}
DJR528	TTTTTTgcggccgCCTCCAAAATCGTCTTCCGCTCTGA	*let-858* 3’UTR
DJR521	TTTTTTggatccGCGATCGCggccggcCTTACTATAAAAAAGTTTGAATACAATTAAATTTC	*let-858* 3’UTR
DJR504	TTTTTTatcgatttaTTTGTATAGTTCATCCATGCCATG	GFP
DJR462	TTTTTTggatccATGAGTAAAGGAGAAGAACTTTTC	GFP
DJR484	AAAAAAAAggccggcctggcATGCAAGCGATCAAATGTGTCGTCG	*ced-10*
DJR485	TTTTTTatcgatTTAGAGCACCGTACACTTGCTCTTTTTGG	*ced-10*
DJR513	GGGATACAGCTGGACTGGAAGATTACGATCGAC	Q61L
DJR514	GTCGATCGTAATCTTCCAGTCCAGCTGTATCCC	Q61L
DJR571	AAAAAAggccggcctggcATGCTGATGCTCACCTTTGCCTC	*egl-1*
DJR572	AAAAAAcccgggTTAAAAAGCGAAAAAGTCCAGAAGACG	*egl-1*
KHP1	GCAAGAGGTCCAAACAGTTCAGAGG	*pkn-1*
KHP2	TGCTTGACTTGGACCAGAACGGTCG	*pkn-1*
KHP3	CCAAGAAGCGTGAGGCCAGAGAAGC	*pkn-1*
KHP4	ACGCCTATGGGGCCACAATGACC	*pes-7*
KHP5	CGATTAAAAAGCAAGCGTACAGGC	*pes-7*
KHP6	ACCTGTGTAGGTGTGAGGAAGTCC	*pes-7*
nv162.f1	ccggcaggaagactatatgactc	*max-2*
nv162.r1	CACAAAGAGGGAAGAAGATCCTC	*max-2*
nv162.r2	CCTTCTTCTGATCGGCAAGACTG	*max-2*
DJR636	ATGAGGGCATGTAATACACAAGTACCG	*pak-1*
DJR637	TTGCATGCTTATTCTCACGCATCACC	*pak-1*
DJR638	GAATCTCTTCCAGGGAAGTCGGG	*pak-1*

Supplementary Protocol

We provide the following protocol to generate reagents that impose conditional lethality. Many of the principles are universal, while some of the tools and reagents discussed here are specific for use in *C. elegans*. The details for conditional expression of toxic proteins are likely to vary in other systems.

Selection of biological processes to target

We targeted CED-10/Rac because of its high level of sequence and functional conservation. We expressed it specifically in epithelia undergoing morphogenesis because we wanted to avoid targeting cell fate decisions or cell proliferation. *The protein/pathway of interest to be targeted by each investigator is likely to be guided by their specific research interests. We emphasize that not all tissues or organisms may be optimally suited to develop this assay. Rather, we suggest that researchers develop assays using whichever system and/or tissue is best suited for the process they wish to target.*

- Proteins whose expression is to be controlled to confer toxicity should be selected based on potential for gain-of-function toxicity, either through constitutive activation through mutation (gain of normal function), mis-expression (gain of novel function), or removal of negative regulator through conditional knockout, including engineered temperature-sensitive mutations[3] or chemogenetic tools like the auxin inducible degron[4-6].
    - Mutational activation of CED-10/Rac via the Q61L mutation described here to disrupt morphogenesis is an example of gain of normal function.
    - Mis-expression of the EGL-1/BH3-only protein in epithelial (hypodermal) cells to induce apoptosis is an example of gain of novel function.
- Tissues to be targeted should be guided based on prior evidence of function in those tissues.

- The case for the morphogenetic hypodermis as target tissue is that it is post-differentiation and post-mitotic[7]. CED-10/Rac, a well-known regulator of cytoskeletal dynamics, has also been validated to play an important role in a series of post-mitotic morphogenetic events in the *C. elegans* mid-embryo[8 9,10].
- Although cells in the embryonic hypodermis do not typically undergo apoptosis[11], we chose to express EGL-1/BH3-only in this tissue so we could compare directly to toxicity conferred by mutationally activated CED-10/Rac, with only the cDNA expressed differing between the two tools. Indeed, we screened through far more candidate transgenes for EGL-1/BH3-only expression than for CED-10/Rac expression, and were never able to consistently obtain 100% lethality.
- A more promising tissue in which to evoke apoptosis through ectopic expression of EGL-1/BH3-only would be neurons, among which a large number undergo apoptosis during the normal course of *C. elegans* development[11,12].

- Piloting toxicity: Prior to assembling the entire system, a quick pilot experiment to evaluate the original premise may increase the likelihood of success. For CED-10/Rac, we overexpressed three small GTPases known from other systems to control cytoskeletal dynamics during cell movements – Rac, Rho and Cdc42[13] – by generating high-copy transgenes with cosmid clones of genome intervals containing each gene. This is a relatively quick assay. Of these, CED-10/Rac conferred diverse defects in morphology with greater penetrance than did Rho/RHO-1 or Cdc42/CDC-42. This was our sole indicator that CED-10/Rac was a promising candidate.

Selection of conditional expression systems

Like selection of proteins of interest, selection of tissues to be targeted should be performed in consultation with the literature and/or an expert in the field to make decisions most likely to result in success.

- The promoter is part of the conditional expression system in our application, driving expression only in a defined tissue. In this context, we selected a specific variant of the *lin-26* promoter, eFGHi, previously demonstrated to express mainly in hypodermal cells during embryonic morphogenesis. The complete range of *lin-26* expression includes post-embryonic epithelial cells as well as diverse support cells, and thus would likely complicate interpretation and make subsequent analysis more difficult[14]. Other promoter types could target other tissues, developmental stages, or environmental conditions, including heat-shock promoters[15].
- Both spatial and temporal control.
    - In addition to the eFGHi variant of the *lin-26* promoter to confer spatial specificity, we coupled conditional degradation through the 3'UTR to confer temporal specificity via temperature sensitive mutations that abrogate NMD. Nonsense-mediated decay has a robust history in *C. elegans*[16] but has the potential downside of regulating many transcripts of the animal, including potentially those that are part of normal regulation of development of genome surveillance, rather than the aberrant premature termination codons for which NMD is best known[17].
    - A similar informational suppressor system uses an intron from the *unc-52* gene that is selectively spliced by MEC-8, a splicing factor for which a temperature-sensitive allele exists. Retention of the intron abrogates gene function, and splicing of the intron relies on *mec-8(u218*ts*)*[18].
    - We point to recent advances with the auxin-inducible degron (AID), a conditional degradation system that requires a substrate protein to be tagged with the AID sequence, requires the

TIR1 co-factor that can be expressed in different tissues or different times, and requires addition of the auxin small molecule to trigger degradation[4]; Ashley *et al.*, in press; preprint available at https://doi.org/10.1101/2020.05.12.090217 .

Generation of transgenes expressing toxic proteins

To engineer toxicity, one must be able to generate tools under conditions that permit viability. As noted in this study, the *smg-1(*ts*)* system is leaky: at 15˚C, protein is expressed and causes some level of toxicity. This feature of the system made it difficult to generate conditionally toxic transgenes in the *smg-1(*ts*)* animals at 15˚C: we systematically biased against the weakly expressing transgenes that could be tolerated by the animal. We therefore developed a protocol to reproducibly isolate toxic transgenes, starting with the wild-type animal in which expression was less leaky.

- Inject the DNA mix, including selection markers ($P_{myo\text{-}2}$*::gfp* and/or *rol-6(*d*)*) into a wild-type background, and isolate scores of independently derived extrachromosomal arrays.
- Plate each candidate line on bacteria expressing dsRNA targeting a gene required for NMD (we used *smg-1*, clone C48B6.6, address I-3K02).
- Select transgenes causing a range of severity of defects when grown on bacteria expressing *smg-1*-directed dsRNA[19]. Score several lines semi-quantitatively (for speed) and forward for further analysis. (We do not freeze the scores of lines, typically settling for ~5 for freezing and further analysis).
- We then integrate extrachromosomal arrays in the wild-type animal background using a variety of published protocols: UV, gamma irradiation, etc.[20].

- Re-test with *smg-1(RNAi)*. Some integrated lines are approximately the same strength, while others are markedly weaker, often accompanied by decreased expression of the P_{myo-2}*::gfp* pharyngeal GFP co-expression marker. We discard the latter.
- Outcross resulting integrants 4x into the wild-type strain background. Freeze the resulting outcrossed strain.
- For candidates of different levels of severity on *smg-1(RNAi)*, cross into the *smg-1(cc546*ts*)* strain, using either SNP-snip PCR detection of the *cc546* lesion (Supplementary Figure 6) or balancing the *smg-1* locus with a mutation in the closely linked *unc-87*. (*N.B.* most strains will display some lethality at permissive temperature of 15˚C).
- Be extremely careful about genetic drift; we observed that strains conferring toxicity became less severe when cultured over many generations.
- To prevent drift, we perform the following steps.
    - Immediately starve and freeze candidate strains. We also test thaw and quantitatively assess lethality to ensure that the frozen strain has not drifted.
    - Maintain the strain as a starved, parafilmed plate for a few months. From this plate, we would extract animals weekly with a chunk of agar, to constantly provide "fresh," undrifted animals for assays or further crosses.
    - Severity can be reset by reconstructing the strains by crossing in *unc-87* to balance *smg-1* and then re-isolating the original strain. Thus, we hypothesize that modifying mutations accumulate over time, though we cannot rule out silencing of the transgene.
- Assess toxicity for each *smg-1(*ts*)*+transgene combination for those reaching 100% lethality. Select for further analysis those that are not at the upper end of the temperature range; above 25˚C, animals can be difficult to culture without drift.

- Generate an efficacy curve at a range of temperatures (T-curve). Procced only with those that confer 100% lethality at or below 25˚ but are fecund at 15˚C
- Validate source of toxicity by RNAi-dependent depletion of the toxic protein, small molecule inhibition, genetic perturbation of "downstream" intermediaries, etc. (see **Fig. 2**).

This protocol should yield strains with properties similar to those described here. However, we note that successfully attaining the desired goal of 100% lethality is a function of multiple variables. Selection of transgenes that confer the strongest defects biases the results towards success, but some systems may not be capable of reproducibly driving 100% lethality. As an example, we use our system with EGL-1/BH3-only expressed conditionally in hypodermal cells. The strain we analyzed was selected from many scores of candidates, and still fell short of reproducibly reaching 100% lethality. (We could attain 100% lethality at 27˚C, but resultant animals were sickly and sterile).

Supplementary Figure Legends

Supplementary Figure 1. Epithelial-specific GFP expression at 15˚C. a-d) The same early enclosure staged *smg-1(cc546*ts*)*; *reIs8[P_{lin-26}::gfp::NMD^{S}3'UTR]* embryo in different focal planes. **a, b)** Epifluorescence (500 msec exposure) and DIC images, respectively, of the dorsal surface of the embryo, with arrows indicating a row of intercalating epithelial cells. **c,d)** Epifluorescence (500 msec exposure) and DIC images, respectively, of a medial section of the embryo, with arrows indicating the column of intestinal cells.

Supplementary Figure 2. NMD-dependent differences in gene expression. Animals harboring the *cc546* temperature-sensitive mutation in *smg-1* have increased *unc-54(r293)* mRNA levels at 25°C by RT-PCR, with *pmp-3* RNA as a control. RNA extractions were performed on pools of adult animals raised at either 15°C or 25°C. cDNA preparations of each strain were subjected to 25, 30 or 35 cycles of PCR with *unc-54*-specific primers. Temperature-dependent differences were visible at 30 cycles with the *cc546*ts allele of *smg-1* used in this study but not the *smg-1(+)* or *smg-1(r861)* putative null mutation, as shown in the graph. Band intensities were quantified using the Image J gel analysis tool. Experiment was performed two times.

Supplementary Figure 3: A *smg-1* temperature-sensitive allele regulates locomotion. All strain backgrounds harbor NMD-sensitive *unc-54(r293*). Photomicrographs were captured from agar plates with 25 msec exposures under same lamp settings. Body posture is representative of locomotion and hence myosin production by the *unc-54* gene and its NMD-sensitive mutation in the *unc-54* 3'UTR, *r293*: deep body bends represent typical locomotion, shallow bends represent flaccid paralysis. **a)** *unc-54(r293)* animals were paralyzed and egg-laying defective (Egl). **b)** The locomotion

and Egl defects of the *r293* mutant were strongly rescued by loss of *smg-1* function. **c)** Locomotion and Egl defects were not as severe with *cc546*ts as with *smg-1(+)* at 15˚C and **d)** are completely suppressed at 25˚C, consistent with *cc546*ts being temperature sensitive.

Supplementary Figure 4: TS NMD-sensitive *unc-97(su110)*. Upon crossing into the reference strain for *unc-97(su110)*, HE110, we observed that the strain contained a background mutation partially suppressing the Unc phenotype of *unc-97(su110)*. Whole genome sequencing of this strain identified a nonsense mutation in *smg-1*, which we named *re1* (see **Supplementary Figure 6**).). Photomicrographs were captured from agar plates with 25 msec exposures under same lamp settings. Body posture is representative of locomotion and hence PINCH production by the *unc-97* gene and its NMD-sensitive mutation in the *unc-54* 3'UTR, *r293*: deep body bends represent typical locomotion, shallow bends represent flaccid paralysis. Arrows point to a clear area posterior to the pharynx that indicates a clear patch in the intestine that indicates distension with liquid due to defective defecation. **a)** *unc-97(su110)* animals alone are paralyzed, Egl, and constipated. **b)** These phenotypes are suppressed by the *smg-1(re1)* mutation crossed back into the *unc-97(su110)* background, **c)** not suppressed by *smg-1(cc546*ts*)* at 15˚C but **d)** suppressed by *smg-1(cc546*ts*)* at 25˚C. Mutants for *unc-97* have been reported to have mechanosensory defects (Chen and Chalfie, 2014), and are thereby sluggish and do not move on plate assays. Consequently, we did not include *unc-97(su110)* in our locomotion analysis for **Supplementary Figure 5**.

Supplementary Figure 5. Quantification of temperature-dependent locomotion regulated by NMD. Putative null mutations in *smg-1* suppress locomotion defects conferred by the aberrant *unc-54(re293)* 3'UTR at both 15˚C and 25˚C. *smg-1(cc545*ts*)* and *smg-1(cc546*ts*)* fail to rescue

locomotion defects at 15˚C but rescue at 25˚C. All animals were scored in sequential assays on the same day, 20-minute assays, then transferred to -20˚C for 5 minutes to arrest locomotion, then counted.

Supplementary Figure 6: Identification of lesions in *smg-1.* The exon-intron boundaries of the *smg-1* gene are shown. Domains are SMG1 (red), PI3Kc (blue) and FATC (gray), UTRs are white. Scale bar = 1000 bp. *cc545*ts is an ACA>ATA transition in exon 15 that causes a T761I missense change. *cc546*ts is an unusual ATG>TTG transversion in exon 35 that causes a M1957L missense change. *re1* is an unusual GAG>TAG transversion in exon 36 that causes an E2093* nonsense change. *cc546*ts is detectable by SNP-snip: restriction enzyme Msl I cuts the wild-type but not the mutant sequence.

Supplementary Figure 7: Alignment of SMG-1 orthologs from different species. Shown is a Clustal Omega alignment on default settings, with a single manual curation to align the C-terminal FATC domain, where the *C. elegans* sequence was likely not aligned due to gap penalties. Sequences are *Drosophila melanogaster* nonC (DmnonC), *Homo sapiens* SMG1 (HsSMG1 and *Caenorhabditis elegans* SMG-1a (CeSMG-1a). Domains predicted by SMART are highlighted with a key at the beginning. The *cc545*ts and *cc546*ts mutations alter conserved residues in the SMG1 (T761I) and PI3Kc lipid kinase (M1957L) domains, respectively (bold, underlined, red).

Supplementary Figure 8. Weak morphogenetic phenotypes. Low penetrance and low expressivity phenotypes are caused by the GFP over-expressing strain *smg-1(cc546*ts); *reIs8[Plin-26::gfp::NMD*^{S}*3'UTR]*, perhaps due to over-represented promoter sequences titrating factors

important for morphogenesis. Arrows indicate mild bulges in the animal’s epithelium, frequently around the head in animals grown at 23˚C. Occasional animals with such bulges grew slower, presumably due to compromised feeding. In this experiment GFP lethality = 0.8% (4/453), WT lethality = 0.6% (4/671).

Supplementary Figure 9. EHT 1864 rescue vs. negative control EHT 8560. 6-well plate assays for small molecule rescue of lethality conferred by *smg-1(cc546*ts*)*; *reIs6* lethality at 23˚C. **Top row:** Synchronized groups of animals treated with increasing doses of negative control molecule EHT 8560. None survived to hatch. (Tracks were left by Rol parents that laid the eggs). **Bottom row:** Synchronized groups of animals treated with increasing doses of Rac inhibitor EHT 1864. Arrows indicate a subset of grown adults, but many smaller larvae are evident. In all images, dark spots are salt crystals in the agar.

Supplementary Bibliography

1 Brenner, S. The genetics of Caenorhabditis elegans. *Genetics* **77**, 71-94 (1974).

2 Reiner, D. J. *et al.* Behavioral genetics of caenorhabditis elegans unc-103-encoded erg-like K(+) channel. *J Neurogenet* **20**, 41-66, doi:10.1080/01677060600788826 (2006).

3 Tan, G., Chen, M., Foote, C. & Tan, C. Temperature-sensitive mutations made easy: generating conditional mutations by using temperature-sensitive inteins that function within different temperature ranges. *Genetics* **183**, 13-22, doi:10.1534/genetics.109.104794 (2009).

4 Zhang, L., Ward, J. D., Cheng, Z. & Dernburg, A. F. The auxin-inducible degradation (AID) system enables versatile conditional protein depletion in C. elegans. *Development* **142**, 4374-4384, doi:10.1242/dev.129635 (2015).

5 Duong, T., Rasmussen, N. R., Ballato, E., Mote, F. S. & Reiner, D. J. The Rheb-TORC1 signaling axis functions as a developmental checkpoint. *Development* **147**, doi:10.1242/dev.181727 (2020).

6 Cho, U. *et al.* Rapid and tunable control of protein stability in Caenorhabditis elegans using a small molecule. *PLoS One* **8**, e72393, doi:10.1371/journal.pone.0072393 (2013).

7 Chisholm, A. D. & Hardin, J. Epidermal morphogenesis. *WormBook*, 1-22, doi:10.1895/wormbook.1.35.1 (2005).

8 Walck-Shannon, E., Reiner, D. & Hardin, J. Polarized Rac-dependent protrusions drive epithelial intercalation in the embryonic epidermis of C. elegans. *Development* **142**, 3549-3560, doi:10.1242/dev.127597 (2015).

9 Soto, M. C. *et al.* The GEX-2 and GEX-3 proteins are required for tissue morphogenesis and cell migrations in C. elegans. *Genes Dev* **16**, 620-632, doi:10.1101/gad.955702 (2002).

10 Patel, F. B. *et al.* The WAVE/SCAR complex promotes polarized cell movements and actin enrichment in epithelia during C. elegans embryogenesis. *Dev Biol* **324**, 297-309, doi:10.1016/j.ydbio.2008.09.023 (2008).

11 Sulston, J. E., Schierenberg, E., White, J. G. & Thomson, J. N. The embryonic cell lineage of the nematode Caenorhabditis elegans. *Dev Biol* **100**, 64-119, doi:10.1016/0012-1606(83)90201-4 (1983).

12 Sulston, J. E. & Horvitz, H. R. Post-embryonic cell lineages of the nematode, Caenorhabditis elegans. *Dev Biol* **56**, 110-156, doi:10.1016/0012-1606(77)90158-0 (1977).

13 Hall, A. Rho GTPases and the actin cytoskeleton. *Science* **279**, 509-514, doi:10.1126/science.279.5350.509 (1998).

14 Landmann, F., Quintin, S. & Labouesse, M. Multiple regulatory elements with spatially and temporally distinct activities control the expression of the epithelial differentiation gene lin-26 in C. elegans. *Dev Biol* **265**, 478-490 (2004).

15 Stringham, E. G., Dixon, D. K., Jones, D. & Candido, E. P. Temporal and spatial expression patterns of the small heat shock (hsp16) genes in transgenic Caenorhabditis elegans. *Mol Biol Cell* **3**, 221-233, doi:10.1091/mbc.3.2.221 (1992).

16 Stalder, L. & Muhlemann, O. The meaning of nonsense. *Trends Cell Biol* **18**, 315-321, doi:10.1016/j.tcb.2008.04.005 (2008).

17 Muir, V. S., Gasch, A. P. & Anderson, P. The Substrates of Nonsense-Mediated mRNA Decay in Caenorhabditis elegans. *G3 (Bethesda)* **8**, 195-205, doi:10.1534/g3.117.300254 (2018).

18 Calixto, A., Ma, C. & Chalfie, M. Conditional gene expression and RNAi using MEC-8-dependent splicing in C. elegans. *Nat Methods* **7**, 407-411, doi:10.1038/nmeth.1445 (2010).

19 Timmons, L., Court, D. L. & Fire, A. Ingestion of bacterially expressed dsRNAs can produce specific and potent genetic interference in Caenorhabditis elegans. *Gene* **263**, 103-112, doi:10.1016/s0378-1119(00)00579-5 (2001).
20 Praitis, V. & Maduro, M. F. Transgenesis in C. elegans. *Methods Cell Biol* **106**, 161-185, doi:10.1016/B978-0-12-544172-8.00006-2 (2011).
