# Supplementary material for: Engineering threshold-based selection systems
Source: G3 (Bethesda). 2021 Jul 14;11(9):jkab234. doi: 10.1093/g3journal/jkab234 (PMC8496214; doi:10.1093/g3journal/jkab234)
Supplement: jkab234_Supplementary_Data [file jkab234_supplementary_data.zip › jkab234-suppl_data/GENETICS-G3-2021-402425-s06.pptx]

## Slide 1
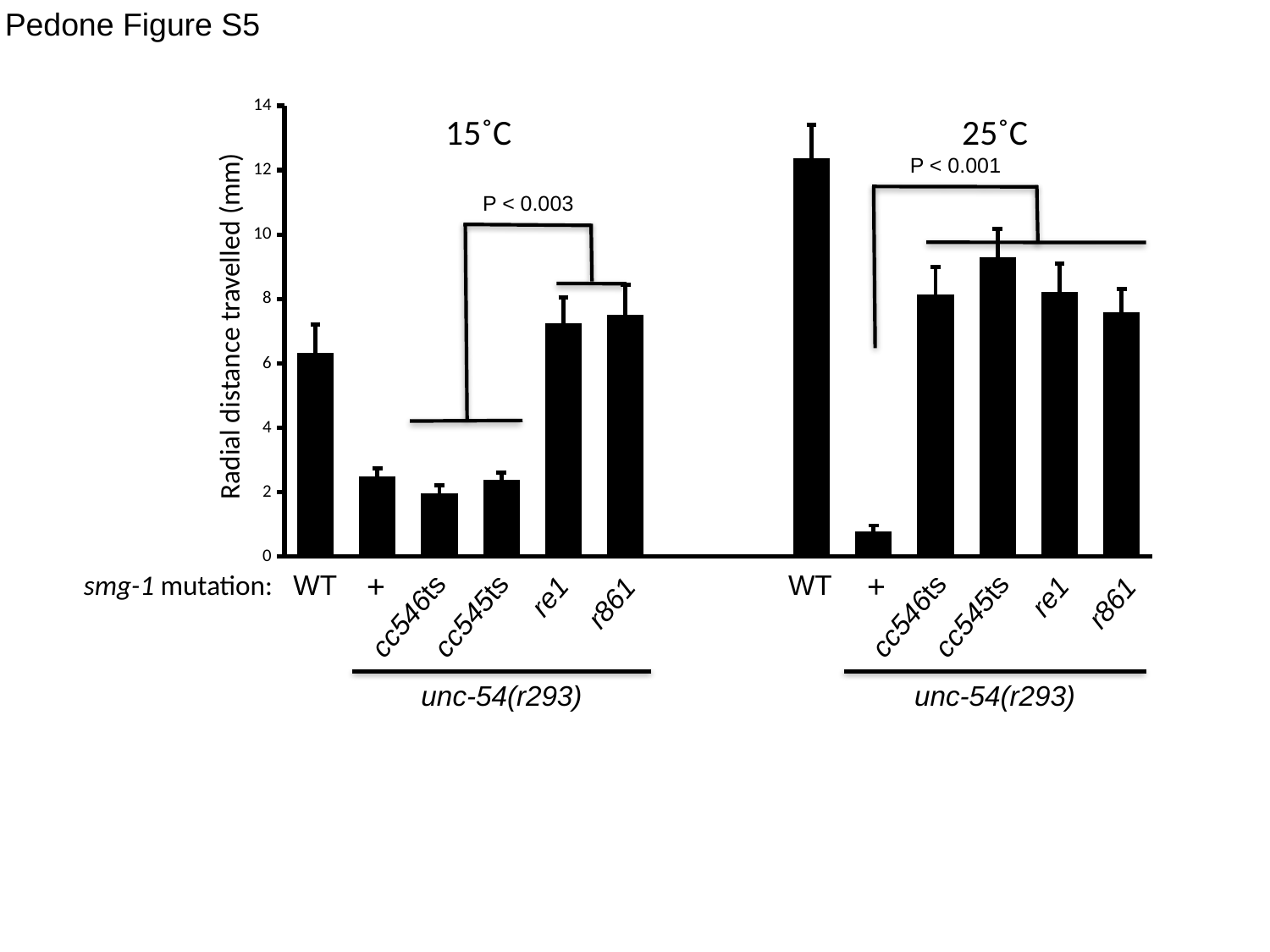

Pedone Figure S5
### Chart
| Category | |
|---|---|15˚C
25˚C
P < 0.001
P < 0.003
Radial distance travelled (mm)
+
smg-1 mutation:
WT
re1
r861
cc546ts
cc545ts
+
WT
re1
r861
cc546ts
cc545ts
unc-54(r293)
unc-54(r293)
