# Supplementary material for: Engineering threshold-based selection systems
Source: G3 (Bethesda). 2021 Jul 14;11(9):jkab234. doi: 10.1093/g3journal/jkab234 (PMC8496214; doi:10.1093/g3journal/jkab234)
Supplement: jkab234_Supplementary_Data [file jkab234_supplementary_data.zip › jkab234-suppl_data/GENETICS-G3-2021-402425-s08.docx]

Pedone Figure S7

Domain codes based on SMART

Green: SMG1

Blue: PI3Kc

Gray: FATC

**Red**: mutated residue

CLUSTAL O(1.2.4) multiple sequence alignment

DmnonC MK--------------------------------NAIHPENCNGAGTE-EEASSAFH--- 24

HsSMG1 MSRRAPGSRLSSGGGGGGTKYPRSWNDWQPRTDSASADPDNLKYSSSRDRGGSSSYGLQP 60

CeSMG-1a ------------------------------------------------------------ 0

DmnonC ---AEIDRVLLNNNGNHGDSSNEGGGGNGSGRGGATGSGNIAGLGGSESMWSPGGGKSHD 81

HsSMG1 SNSAVVSRQRHDDTRVHADIQNDEKGGYS-----------------------VNGGSGEN 97

CeSMG-1a ------------------------------------------------------------ 0

DmnonC V--AQAFANALLLRNMNHVVGKGQPVVQNHRKAY---QCKGDTINPMANGEDLRLSKIIR 136

HsSMG1 TYGRKSLGQELRVN---NVTSPEFTSVQHGSRALATKDMRKSQERSMSYSDESRLSNLLR 154

CeSMG-1a ----------------------------------------------MITSRNNDIGNLIE 14

* . : :.:::.

DmnonC RLINENNPT-VSLELCSKLDQAVRTPINMGYMTCSFVWILDNMLT--LYKQCPPPVLEEC 193

HsSMG1 RITREDDR-DRRLATVKQLKEFIQQPENKLVLVKQLDNILAAVH-DVL-NESSKLLQELR 211

CeSMG-1a QFRQRDTPQKERKAILARIEEILQTTKNVESLCVKWTYLLDNLCWPSLTKHDRNDMKTLA 74

:: ..: ::.: :: * : . :* : * :. :

DmnonC SKTLGLIGFINRK---SYPIYEEFIVKNYKSSKRMQKYMIMAL----------------- 233

HsSMG1 QEGACCLGLLCASLSYEAEKIFKWIFSKFSSSAKDEVKLLYLCATYKALETVGEKKA--- 268

CeSMG-1a GKVIRLVGV----LLFDTESY--------------PEFLIYLGTLYQSVTKKSEETRADI 116

: :*. . ::

DmnonC -------RATLSCDTKCEL-HMYADKIMLLLKDFLEN--AESADIFIVVSNTLVQFAASY 283

HsSMG1 -F------------------SSVMQLVMTSLQSILEN--VDTPELLCKCVKCILLVARCY 307

CeSMG-1a VFSVYFIVGVISQKTENRLIATDTENVEKSLDWIIKVLPNSSISVYNHCLKGFVLVANTF 176

: : *. ::: .: .: : :: .* :

DmnonC AETFECHFTDVVDIVIGWQLEAGQPTDLKTHCAQVLEQLTPFFSKQIDFSYGLLDQFVED 343

HsSMG1 PHIFSTNFRDTVDILVGWHIDHTQKPSLTQQVSGWLQSLEPFWVADLAFSTTLLGQFLED 367

CeSMG-1a PNVYAAMFESTLRAILTNLPDFNS------HEKNF----ELLIDTVMRFSDQ-----LNE 221

. : * ..: :: : . : : : ** :::

DmnonC ITTLEEGE---------------PANTAERVGAFVGAFNTLLKCLA-RMQIFVGMPTCEC 387

HsSMG1 MEAYAEDLSHVASGESVDEDVPPPSVSLPKLAALLRVFSTVVRSIGERFSPIRGPPITEA 427

CeSMG-1a KPHLAEEMVRII----------RPDIKKNGLGNM--------RELKKRM------KLTMA 257

* * . :. : : : *: .

DmnonC IVKMAVDHLIKIMPTLHL--NTEALVNI-N--------------ELICICLLN-NFTGLD 429

HsSMG1 YVTDVLYRVMRCVTAANQVFFSEAVLTAANECVGVLLGSLDPSMTIHCDMVIT---YGLD 484

CeSMG-1a LVKM--AKSQKMLEETNQMI-SEMSIEL-EENGGK-W-SSASLITIVCDVFNELLILGKD 311

*. : : : : :* : : : * . * *

DmnonC PILLEQVLLDQVK------RMISLTELQRQSVLYLLLCTVRRLRARLT-PSLVHFIFQSN 482

HsSMG1 QLENC-----QTCGTDY-----------IISVLNLLTLIVEQINTKLP-SSFVEKLFIPS 527

CeSMG-1a DVKLQKGVEESLCNVLKDLNLSNQSTMEKQAFFNSLAKIVKQLPAESQVKTRVHQIVFNT 371

: . :.: * *.:: :. : *. :. .

DmnonC ------PYMTKVRLRSPGETSYKLLLRTCQETLLIRNVPLLQQAYKYLVDDIDACLEKLL 536

HsSMG1 SKLLFLRYHKEKEVVAVAHAVYQAVLSLKNIPV-------LETAYKLILGEMTCALNNLL 580

CeSMG-1a ETGLFTPKNRDNRM-FGHNMIYKDLINLVSVLLTPTSLNHLQATYTDLRKIMIDS----- 425

. .: . *: :: . : *: :*. : : .

DmnonC IT----------------APRSKARKASVLLVFHLSALAALAKQTSSIIGMYACKPSILE 580

HsSMG1 HSLQLPEACSEIKHEAFKNHVFNVDNAKFVVIFDLSALTTIGNAKNSLIGMWALSPTVFA 640

CeSMG-1a --------MSRLKQSEDTPYSDNIRWNESILLLFFSSLQSISCAKSSLIVMMGIRPSIFE 477

: . :::: :*:* ::. ..*:* * . *:::

DmnonC LLLTNCRAHELKFWSKYPAAQQAIFGLLVVHCQANHNFRTNSSL-----LRDQELSAENT 635

HsSMG1 LLSKNLMIVHSDLAVHFPAIQYAVLYTLYSHCTRHDHFISSSLSSSSPSLFDGAVISTVT 700

CeSMG-1a FFSSELPLTEYWLASNHPEVYHLFITIFVGHLKAHDFYIVQSD-----YIVRGDSIGQSI 532

:: .: . : :.* .: : * :. : .* : .

DmnonC SPTANSFA--------SILRFLDSVLGQAHQLAPQNLRVLLQWIQMLLRE-CRE-KIDLL 685

HsSMG1 TATKKHFS--------IILNLLGILL-KKDNLNQDTRKLLMTWALEAAVLMKKSETYAPL 751

CeSMG-1a GQTKRDYARKQVVALQKIINNFG------DKLWKKTRLMISSWLHSLIATACEHQIGSDS 586

* . :: *:. :. .:* .. :: * .

DmnonC MEQENFRGICRNIAATASKLVPLESAACIQTVLDYGLERLEKYPKLLILYRD-------- 737

HsSMG1 FSLPSFHKFCKGLLANTL---VEDVNICLQACSSL--------HALSSSLPDDLLQRCVD 800

CeSMG-1a FSQREWVRLRNTVIHQSV---LTWNNECVNQALTIL-STATKWSELTSDIHRDIADKTKK 642

:. .: : . : : *:: *

DmnonC ------TALQQLQMLSTNYHAPYFQIYAQLPLHLTLTGGESSMPG---MASRRV----SV 784

HsSMG1 VC---RVQLVHS---GTRIRQAFGKLLKSIPLDVVLSNNNHTEIQEISLALRSHMSKAPS 854

CeSMG-1a AKWKEATTIWESGDCNTYIRQSMSTVY--------------------QMSQERQQKTITS 682

. : . .* : : :: .

DmnonC WQQRISQYSAVRDNVFRDFFDRVQKPEQDSLIHCLRELFVRSCQVAPQDERQMNLSQCTK 844

HsSMG1 NTFHPQDFSDVISFILYG---NSHRTGKDNWLERLF----YSCQRLDKRDQS-TIPRNLL 906

CeSMG-1a TSFGAEEFIIITNFLLKQATPTTFKKGQNSWMDEVLETFTQGCRTLEKPDS--YVPE--- 737

.:: : . :: : ::. :. : .*: : : : .

DmnonC RCQRLAIAWLQFEAARYCVDQRLRTTVGKPQETFLGFEAIIMRHARLLSGCAKE---IER 901

HsSMG1 KTDAVLWQWAIWEAAQFTVLSKLRTPLGRAQDTFQTIEGIIRSLAAHTLNPDQDVSQWTT 966

CeSMG-1a -TFIEKWDWIINQTANFCIVNKMK**T**PLGKPMQTFAAFENEIKRLAKEVIVRKNSDKKLNK 796

* ::*.: : .:::* :*: :** :* * * :.

DmnonC SALDDLSLE---ELLSMQSNLSLLLGFLDALEKLIYNAAEGSA--FALRPPEKQVAAFFR 956

HsSMG1 ADNDE-------GHGNNQLRLVLLLQYLENLEKLMYNAYEGCAN--ALTSPPKVIRTFFY 1017

CeSMG-1a SSTEDPNQSPPLKYSVQWLRVHLLLKLIVVLEKLMNSAIHGGSSVFNLTEIPVSSRQFFT 856

: :: .: *** : ****: .* .* : * **

DmnonC LNNPTCQSWFNRIRIGVVIIAMHVQQPELVIRYAQQILVNSKTQDPTYSQAI-------- 1008

HsSMG1 TNRQTCQDWLTRIRLSIMRVGLLAGQPAVTVRHGFDLLTEMKTTSLSQGNELEVTIMMVV 1077

CeSMG-1a VNAASCEVWLNRVYYPALLVAYFNGYYGLVIRFGSNALSHFARQKDGDNDK------KIV 910

* :*: *:.*: : :. :.:*.. : * . . .:

DmnonC ------VYMAWSLVSCQEADSLRGLRLWARGKSCKSY--KWLKYAADQAAGKRESALAGY 1060

HsSMG1 EALCEL----------HCPEAIQGIAVWSSSIVGKNL--LWINSVAQQAEGRFEKASVEY 1125

CeSMG-1a NGVCTASLMSLSMAVLGEPMEIVGLRRKVREEFGTDMGQSLMEALGEMANARYETALVAL 970

: *: .. :: .: * .: *.* .

DmnonC RTILAEKELQSELE----------------------------------------PHTRQF 1080

HsSMG1 QEHLCAMTGVDCCISSFDKSVLTLANAGRNSASPKHSLNGESRKTVLSKPTDSSPEVINY 1185

CeSMG-1a EAVLVTDAATN----ETLKMIIQLAM----------------------------VDILNR 998

. * . . :

DmnonC VVSQMMQCLQDLGQWSQLVELKQQQMTRPEDRELNPFLQRSNVEVNALERLLAKSEESCS 1140

HsSMG1 LGNKACECYISIADWAAVQEWQNAIHDLK------------------------------- 1214

CeSMG-1a IRLPQATDYYKV------VLFGE------------------------------------- 1015

: .: :

DmnonC SMDALGGVFQQLSLWPSNWDESVSSSGLSERASFSSIHMRQRTE---------------- 1184

HsSMG1 --------------------KSTSSTSLNLKADFNYIKSLSSFESGKFVECTEQLELLPG 1254

CeSMG-1a -----------------------ESNDSTITEDFRSVELLTKFEKL-------------- 1038

.*.. . .* :. *

DmnonC ------------DIVLHKLLED---------------------RCVPDQAKN-LLDTQWR 1210

HsSMG1 ENINLLAGGSKEKIDMKKLLPNMLSPDPRELQKSIEVQLLRSSVCLATALNPIEQDQKWQ 1314

CeSMG-1a ----------------------------------------------NNTVNEKRQVVDWS 1052

: .*

DmnonC DSLL----NPSFDQRSCKELTLLRH-IVQGVSGGQELSLLPVSSG-----RCQNR----- 1255

HsSMG1 SIT------ENVVKYL-KQ-------TSRIAIGPLRLSTLTVSQSLPVLSTLQLYCSSAL 1360

CeSMG-1a ARERFQFVESAFSQTM-RRTELLDIQKDFSAMGALALSADS---------SCKLYSDI-- 1100

. : :. . * ** :

DmnonC ------------------SKFISSAILMRCLAWTQLLRQHCAPGS--------------- 1282

HsSMG1 ENTVSNRLSTEDCLIPLFSEALRSCKQHDVRPWMQALRYTMYQNQLLEKIKEQTVPIRSH 1420

CeSMG-1a --------SSTSLIIANL-----VNKMTGVSQWKNKLTDTEIFDRNEEGNDGDKLAICRK 1147

* : * .

DmnonC WETLCLDAAAAAREEGNLQLAETLLTQFFGQPIGEIAAL-----FSLEQGVQTDNPEMLR 1337

HsSMG1 LMELGLTAAKFARKRGNVSLATRLLAQCSEVQLGKTTTAQDLVQHFKKLSTQGQVDEKWG 1480

CeSMG-1a LMHWGRHTK---SNRGQSCAAH-----SEIIRLSRKTSNCELAFFHINSAIRGEKLAAWQ 1199

: :.*: * :.. :: . : . : :

DmnonC GYSEL--VKCLHLQQQQSQTHSGDLSSSIDVCAAL-CLNIQKSNNQPAAGADLLLNLADW 1394

HsSMG1 PELDIEKTKL--LYTAGQSTHA---MEMLSSCAISFCK----SVKAEYAVAKSILTLAKW 1531

CeSMG-1a R-LEVERQRLKLVKTQNLDVRIREMNEVFGSLAEVFTTSC--QLKSDFQMVDGMIK-EKM 1255

:: : : ..: . :. * . : .. ::. .

DmnonC IAVRTCNGL-----------------------------TTNQSPVLIQLLDQLPECPLTC 1425

HsSMG1 IQAEWKE-ISGQLKQVYRA--QHQQNFTGLSTLSKNILTLIELPSVNTMEEEYPR--IES 1586

CeSMG-1a ISEGYNEDIAKREEHMSRASIQLADFFQSLPELE-NVLAPNLFPTII-----WSE--LQR 1307

* : : : * : . :

DmnonC DSSQPLAIPQAERMVARLVHSCLQQRPNYAEALIAYGNWCYRWGKKVADSCCVLTQADAT 1485

HsSMG1 ESTVHIGVGEPDFILGQLYHLSSVQAPEVAKSWAALASWAYRWGRKVVDNASQGEGVRLL 1646

CeSMG-1a R-SDSL-SAGYHGIVGSLFHLASEMCPSLAKAHLKMARWAYEIAKIKNF----------- 1354

: : . ::. * * . *. *:: . *.*. .:

DmnonC ---AISQALDIPQPLESEKLDELLQALS---TEQPPANCV-EVCPDAARARDDE------ 1532

HsSMG1 PREKSEVQNLLPDTITEEEKERIYGILG-QAVCRPAGIQDEDITLQITESEDNEEDDMVD 1705

CeSMG-1a PAENLSFYKFGKDE---TENEELLKSLEATSLVNLEKL------VRAAISDDLRA----- 1400

. : : :.: * . : : * .

DmnonC -AAKNRLRRLTFLADKTPEALDAILQIWRRAIANTYDYYKDAARSYFQYLSFKSGSGPEK 1591

HsSMG1 VIWRQLISSCPWLSELDESATEGVIKVWRKVVDRIFSLYKLSCSAYFTFLKLNAGQIPLD 1765

CeSMG-1a --------------NNILAPNSHFMHIWKMVRDHRTKFLSIAVTSYFQFIQNMSGDCD-- 1444

: . .:::*: . . . . : :** ::. :*.

DmnonC PEGEGVVSQRERLHVDDSNLVTTTLRLLRLIVKHASGLQEVLEQGLHTTPIAPWKVIIPQ 1651

HsSMG1 EDDPRLHLSHRVEQSTDDMIVMATLRLLRLLVKHAGELRQYLEHGLETTPTAPWRGIIPQ 1825

CeSMG-1a ----------NLPYSKKEETTLATLRILELLVKHGDVLIDVINDGLNKTNVHIWKEILPQ 1494

. .. . :***:*.*:***.. * : ::.**..* *: *:**

DmnonC LFSRLNHHEPYVRKSVCDLLCRLAKSRPQLVIFPAVVGANREQQDATA------------ 1699

HsSMG1 LFSRLNHPEVYVRQSICNLLCRVAQDSPHLILYPAIVGTISLSSESQASGNKFSTAIPTL 1885

CeSMG-1a LFARLSHPSEHIRKTLVDLISKICTAAPHAVVFQVVSGAASSSTDG-------------- 1540

**:**.* . ::*::: :*:.::. *: ::: .: *: . :.

DmnonC ------------------PPATA------------RPTTEDACCYGYLLGELSKQAPEAV 1729

HsSMG1 LGNIQGEELLVSECEGGSPPASQDSNKDEPKSGLNEDQAMMQDCYSKIVDKLSSANPTMV 1945

CeSMG-1a ---------------------------EELEEQQNDDRNRVRACCEKLETNMSQSYPNLV 1573

* : ::*. * *

DmnonC QHVKLMVKELRRVCLLWDEYWIHSLAHIYNTYVSRVSALATDFRPDDH---EGKNNRF-- 1784

HsSMG1 LQVQMLVAELRRVTVLWDELWLGVLLQQHMYVLRRIQQLEDEVKRVQNNNTLRKEEKIAI 2005

CeSMG-1a KDVRQFVAELERINLLNEEKWSVVMGTMEHEMEKRLSLIRTENAKTESALHLTASVKNDI 1633

.*: :* **.*: :* :* * : *:. : : : . :

DmnonC -----NVWRPQLLADLEA-LVAVTSRPPETTYERSFRKRFDAPIRLTVDALR----HRRY 1834

HsSMG1 MREKHTALMKPIVFALEHV-RSITAAPAETPHEKWFQDNYGDAIENALEKLKTPLNPAKP 2064

CeSMG-1a IVKRTQLLTRQIFDVLDELYQQTVIEPPKSKNEEEFVTAFAEVLTNAFQESRI-SRTTSP 1692

:. *: . * :: *. * : : :.: :

DmnonC PEAWDKLKQLYHILQSNMIRGSGSTLKMQSISPVLCGIGRMRISMPGLDAHGPDGDQVYI 1894

HsSMG1 GSSWIPFKEIMLSLQQRAQKRASYILRLEEISPWLAAMTNTEIALPGEVSA---RDTVTI 2121

CeSMG-1a EKSWIPFKNLIANFVHRNSKKGMQTFETEDISPYLASLSNSCVPMPGQESVE-FDRVVSI 1751

.:* :*:: : . : . :. :.*** *..: . : :** : * *

DmnonC ESVESSVCVLPTKTKPKKVAFYGSNGQRYTFLFKGMEDLHLDERIMQFLSISNAIMACRS 1954

HsSMG1 HSVGGTITILPTKTKPKKLLFLGSDGKSYPYLFKGLEDLHLDERIMQFLSIVNTMFATI- 2180

CeSMG-1a SRVARQVTILPTKTRPKKLGFVGSDGKQVAFLFKGREDLHLDERVMQFLRLCNVMLQPGK 1811

* : :*****:***: * **:*: :**** ********:**** : *.::

DmnonC --DAPGNGCYRAHHYSVIPLGPQSGLISWVDGVTPVFALYKKWQQRRSQVAGNAGAGA-- 2010

HsSMG1 --NRQETPRFHARHYSVTPLGTRSGLIQWVDGATPLFGLYKRWQQREAALQAQKAQDSYQ 2238

CeSMG-1a GKHRQSVAAYQAHHYAVIPLGPRSGLIKWVEGATPMFHIYRKWQMKEKALKQATKKNGET 1871

. ::*:**:* *** :****.**:*.**:* :*::** :. : ..

DmnonC ---VANVPRRFTDLFYNKLSPLLAKHNM--QVSDPRRQWPISVLLQVLDELSQETPNDLL 2065

HsSMG1 TPQNPGIVPRPSELYYSKIGPAL--KTVGLSLDVSRRDWPLHVMKAVLEELMEATPPNLL 2296

CeSMG-1a VP----EIERPSNMYHNMIRLAFADHKIDSSITSDRSKWPAEILEEVFESLTAKTPTDLI 1927

* :::::. : : :.: .: * .** :: *::.* ** :*:

DmnonC ARELWCQAGNAAEWRQSVRRFVRCMSVMSMIGYVIGLGDRHLDNVLINLGSGDIVHIDYN 2125

HsSMG1 AKELWSSCTTPDEWWRVTQSYARSTAVMSMVGYIIGLGDRHLDNVLIDMTTGEVVHIDYN 2356

CeSMG-1a SRELWMRANDATTWWSVTKRYSRSLAVMS**M**VGSVLGLGDRHLDNLLVDLKWGHVVHIDYN 1987

::*** . * .: : *. :****:* ::*********:*::: *.:******

DmnonC VCFEKGRTLRIPEKVPFRLTQNLVQAMGITGIEGPFRLGCEYVLKVMRKERETLLTLLEA 2185

HsSMG1 VCFEKGKSLRVPEKVPFRMTQNIETALGVTGVEGVFRLSCEQVLHIMRRGRETLLTLLEA 2416

CeSMG-1a ICFDKGKNLRIPETVPFRLTRNMRHALGPSEMYGTFRESCVHVLSTLRSGHQVLTMLLDA 2047

:**:**:.**:**.****:*:*: *:* : : * ** .* ** :* ::.* **:*

DmnonC FVYDPLVDWTTNDDAQALRRSLNAKLQESADGGGAGGLGVGDLKYHKKDKNKGKPLDSDV 2245

HsSMG1 FVYDPLVDWTAGGEAGFAG-------AV--YGGGGQ---------QAESKQSKREMEREI 2458

CeSMG-1a FVFDPLVDWTSHEHTATSGVSLALQLAV--YGSNWK---------TKAKER------LTD 2090

**:*******: .: *.. .:.

DmnonC KRQPFLSKLGMLQKYWSTNKTELMPQLEEMEQEVGNLQAAQAKQVVA---------EEEL 2296

HsSMG1 TRSLFSSRVAEIKVNWFKNRDEMLVVLPKLDGSLDEYLSLQEQLTDVEKLQGKLLEEIEF 2518

CeSMG-1a AMELLNLRMSEVQTLWLANRDDLLHWMKQVTECLL----IENSMLGANAIY----AQQRV 2142

. : ::. :: * *: ::: : :: : : . . : ..

DmnonC V---------------KLNQRSALIAEIKSLGTAIE--------SHSFNTASLRNAVRRG 2333

HsSMG1 LEGAEGVDHPSHTLQHRYSEHTQLQTQQRAVQEAIQVKLNEFEQWITHYQAAFNN----- 2573

CeSMG-1a KAG--------TELREAVTRHHALAKELRPLIRVIGKEREEFADYLKFYKQALFDPLLKG 2194

..: * : : : .* .. :: :

DmnonC HSEALALLSTERLPDFGRVQCILRSYGQCLQLYHLLDLQGQLVKLQMESNSENAR----- 2388

HsSMG1 --------------------------------------------LEATQLASLLQEISTQ 2589

CeSMG-1a HSALRNELDI--------DTCVY---NFNIVMQNIDNVFGALVNLSFTPIETITSR-TSQ 2242

*.

DmnonC -EFS-----ALTEALQLSGLDSMRSQLNELLGRMDMVAQKSS--------------KHLQ 2428

HsSMG1 MDLGPPSYVPATAFLQNAGQAHLISQCEQLEGEVGALLQQRRSVLRGCLEQLH------- 2642

CeSMG-1a QEFKPPPGLENVWVVK---------QDQQENSQAREVVRRVERRLNGWLDG--------- 2293

:: . :: * :: .. : ::

DmnonC EYAGV---MNFYPE---QSHRQNLFVRFHDSFATY-----IQNGYTADSTTNTNSPSSSI 2477

HsSMG1 HYATV---ALQYPKAIFQKHRIEQWKTWMEELICNTTVERCQELYRKYEMQYAPQP-PPT 2698

CeSMG-1a ------------------------------------------------------------ 2322

. . .: : : :

DmnonC ICKADVVGVAEAMEYSWERLGCQL----------------------------HEASKLYA 2509

HsSMG1 VCQFIT-----ATEMTLQRYAADINSRLIRQVERLKQEAVTVPVCEDQLKEIERCIKVFL 2753

CeSMG-1a ------------------------------------------------------------ 2322

DmnonC ANQAQALTLGAPTTALLSMIVQSGCSQLLLKASLVRTLDRAGGAFAAYEQ-VALASHDDG 2568

HsSMG1 HENGEEG-----SLSLASVIISALCTLTR------RNLMMEGAASSAGEQLVDLTSRDGA 2802

CeSMG-1a ------------------------------------------------------------ 2322

DmnonC LL--------------------------------------------------------HH 2572

HsSMG1 WFLEELCSMSGNVTCLVQLLKQCHLVPQDLDIPNPMEASETVHLANGVYTSLQELNSNFR 2862

CeSMG-1a ------------------------------------------------------------ 2322

DmnonC QLLFIHLVRTMLQGVLVMTKE---EDQHLA------QLESLLSALSHLKK----MFEYDL 2619

HsSMG1 QIIFPEALRCLMKGEYTLESMLHELDGLIEQTTDGVPLQTLVESLQAYLRNAAMGLEEET 2922

CeSMG-1a ------------------------------------------------------------ 2322

DmnonC PAN---LYR------LLLLQPNLGKLSALCHLSASSLAQLFLEATMENGHKPPDQFPVER 2670

HsSMG1 HAHYIDVARLLHAQYGELIQPRNGSVDETPKMSAG------------------------Q 2958

CeSMG-1a ------------------------------------------------------------ 2322

DmnonC RFLLTLQPVYDQFLLASTSLDSLVSSMQSMLEDV-----HDVQTQQIMELGLM-RSCHTE 2724

HsSMG1 MLLVAFDGMFAQV---ETAFSLLVEKLNKMEIPIAWRKIDIIREARSTQVNFFDDDNHRQ 3015

CeSMG-1a ------------------------------------------------------------ 2322

DmnonC LNDECFF-----------G---LVSEALESS------------------------RTCDV 2746

HsSMG1 VLEEIFFLKRLQTIKEFFRLCGTFSKTLSGSSSLEDQNTVNGPVQIVNVKTLFRNSCFSE 3075

CeSMG-1a ------------------------------------------------------------ 2322

DmnonC REMARPMLGFIHRLQVEKLAGL----LPILTRNFYTAVGPQCLPTASCG-----DPAQAD 2797

HsSMG1 DQMAKPIKAFTADFVRQLLIGLPNQALGLTLCSFISALGVDIIAQVEAKDFGAESKVSVD 3135

CeSMG-1a ------------------------------------------------------------ 2322

DmnonC HLCESLFISLQSDGALLQ---QQAEI--------------------ALLSQQVDLH--TL 2832

HsSMG1 DLCKKAVEHNIQIGKFSQLVMNRATVLASSYDTAWKKHDLVRRLETSISSCKTSLQRVQL 3195

CeSMG-1a ------------------------------------------------------------ 2322

DmnonC AASAQYWAYSEALGSQLRCGPHIVSRPKLTAAIGECWLELDQKLTALQQLQAGLES---Q 2889

HsSMG1 HIAMFQWQHEDLLINRPQ--AMSVTPPPRSA----ILTSMKKKLHTLSQIETSIATVQEK 3249

CeSMG-1a ------------------------------------------------------------ 2322

DmnonC ---LSQLQTQRSNWNRNHIDNLLRMEQCNKQRTMSHVALLQKMTDGAGAVARLEQNAIV- 2945

HsSMG1 LAALESSIEQRLKWAGGANPALAPVLQDFEATIAERRNLVLKESQRASQVTFLCSNIIHF 3309

CeSMG-1a ------------------------------------------------------------ 2322

DmnonC ------------VGEEGQALVDHLEQWLAAHGQWQASSSRIS--AVEQSMVELLDPEGAI 2991

HsSMG1 ESLRTRTAEALNLDAALFELIKRCQQMCSFASQFNSSVSELELRLLQRVDTGLEHPIGS- 3368

CeSMG-1a ------------------------------------------------------------ 2322

DmnonC DHYWLENVQGLLEEQTCKVHREIAAIEGEQQSKHRFICTLLKETLR-------------- 3037

HsSMG1 -SEWLLSAHKQLTQDMS-T---QRAIQTEKEQQIETVCETIQNLVDNIKTVLTGHNRQLG 3423

CeSMG-1a ------------------------------------------------------------ 2322

DmnonC ----LLENMPRFHVQSLC------------------------------SEAQAQGQGKM- 3062

HsSMG1 DVKHLLKAMAKDEEAALADGEDVPYENSVRQFLGEYKSWQDNIQTVLFTLVQAMGQVRSQ 3483

CeSMG-1a ------------------------------------------------------------ 2322

DmnonC -EYANVQLLSDHLREGQGLMQSLYMRLQELRKDI-------CSDR---RVLQPSMLQNWR 3111

HsSMG1 EHVEMLQEITPTLKELKTQSQSIYNNLVSFASPLVTDATNECSSPTSSATYQPSFAAAVR 3543

CeSMG-1a ------------------------------------------------------------ 2322

DmnonC HQL------EMILTLAKQEVNEFFKGLE----DFMQHAGETDSYE-IFTHAKGSGNVHEQ 3160

HsSMG1 SNTGQKTQPDVMSQNARKLIQKNLATSADTPPSTVPGTGKSVACSPKKAVRDPKTGKAVQ 3603

CeSMG-1a ------------------------------------------------------------ 2322

DmnonC KRNAYGVSVWKKIRMKLEGRDPDSNQRSTVAEQVDYVIREACNPENLAVLYEGWTPWV 3218

HsSMG1 ERNSYAVSVWKRVKAKLEGRDVDPNRRMSVAEQVDYVIKEATNLDNLAQLYEGWTAWV 3661

CeSMG-1a --------------------SAGPDRKLSPREEADILIAEATSTPNLSQMYEGWTAWV 2322

.: .:: :: *.:*::* **:: ** :********
