# Supplementary material for: Engineering threshold-based selection systems
Source: G3 (Bethesda). 2021 Jul 14;11(9):jkab234. doi: 10.1093/g3journal/jkab234 (PMC8496214; doi:10.1093/g3journal/jkab234)
Supplement: jkab234_Supplementary_Data [file jkab234_supplementary_data.zip › jkab234-suppl_data/GENETICS-G3-2021-402425-s11.pptx]

## Slide 1
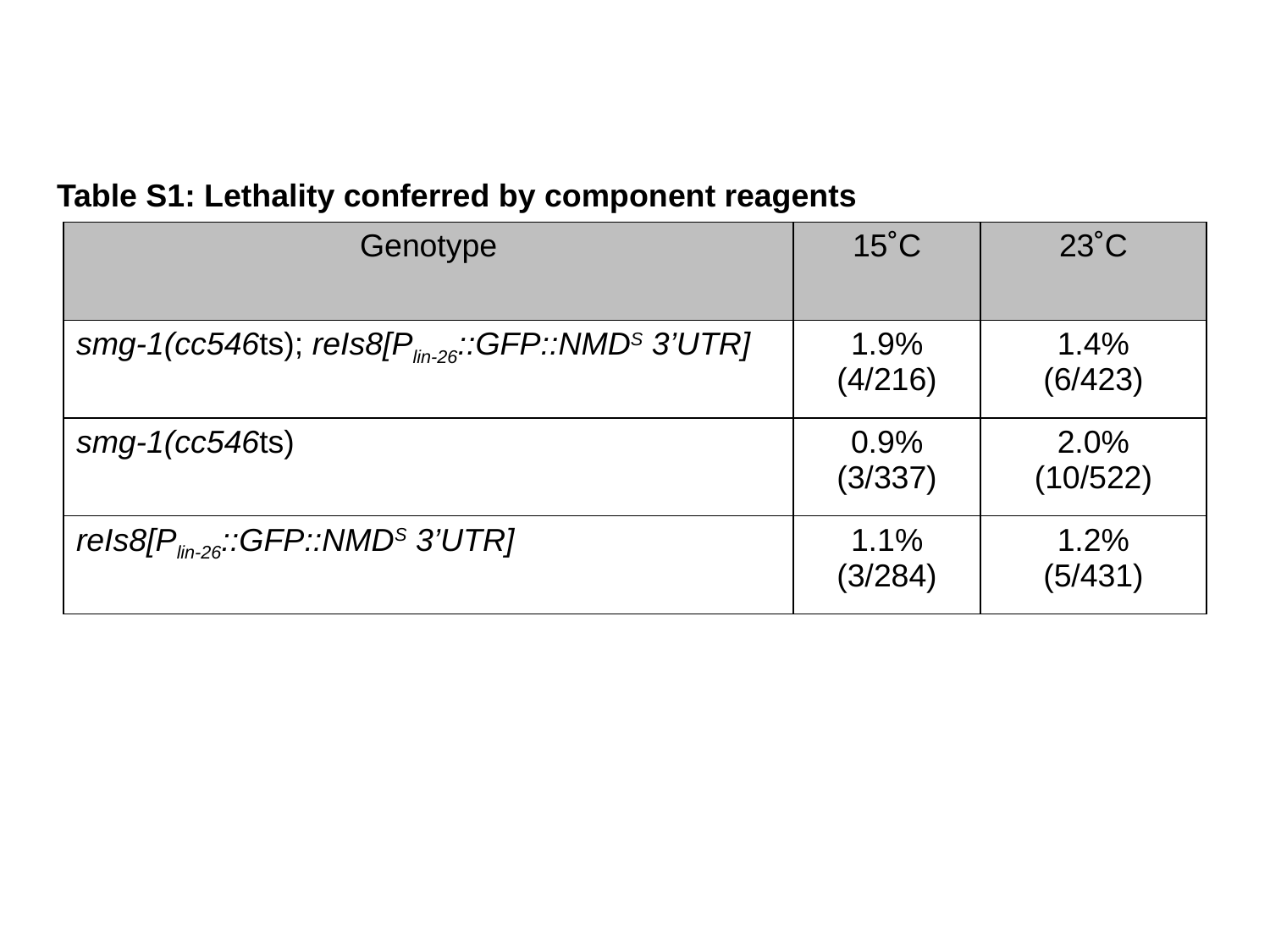

# Table S1: Lethality conferred by component reagents
| Genotype | 15˚C | 23˚C |
| --- | --- | --- |
| smg-1(cc546ts); reIs8[Plin-26::GFP::NMDS 3’UTR] | 1.9% (4/216) | 1.4% (6/423) |
| smg-1(cc546ts) | 0.9% (3/337) | 2.0% (10/522) |
| reIs8[Plin-26::GFP::NMDS 3’UTR] | 1.1% (3/284) | 1.2% (5/431) |
