# Supplementary material for: Engineering threshold-based selection systems
Source: G3 (Bethesda). 2021 Jul 14;11(9):jkab234. doi: 10.1093/g3journal/jkab234 (PMC8496214; doi:10.1093/g3journal/jkab234)
Supplement: jkab234_Supplementary_Data [file jkab234_supplementary_data.zip › jkab234-suppl_data/GENETICS-G3-2021-402425-s12.pptx]

## Slide 1
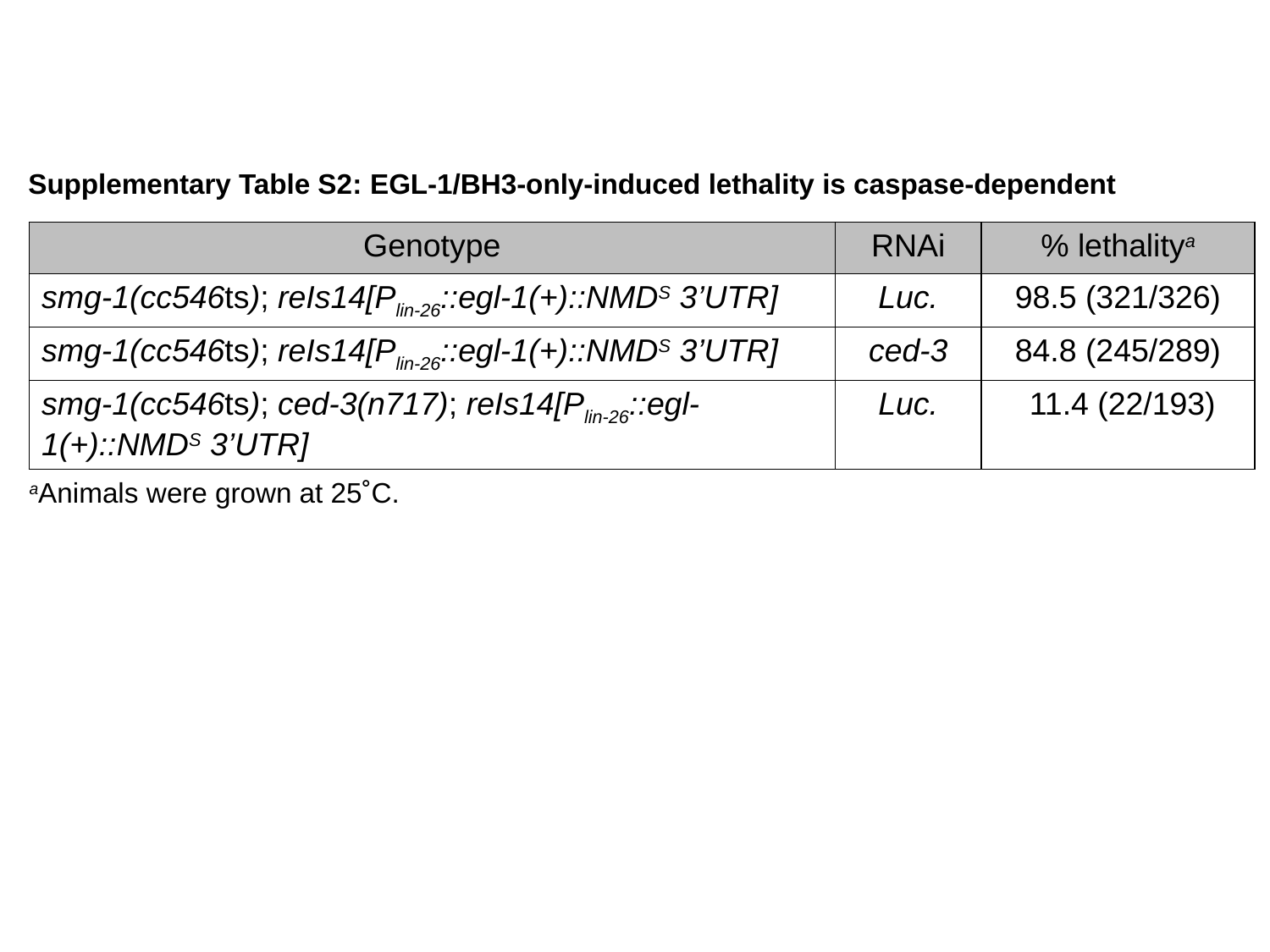

Supplementary Table S2: EGL-1/BH3-only-induced lethality is caspase-dependent
| Genotype | RNAi | % lethalitya |
| --- | --- | --- |
| smg-1(cc546ts); reIs14[Plin-26::egl-1(+)::NMDS 3’UTR] | Luc. | 98.5 (321/326) |
| smg-1(cc546ts); reIs14[Plin-26::egl-1(+)::NMDS 3’UTR] | ced-3 | 84.8 (245/289) |
| smg-1(cc546ts); ced-3(n717); reIs14[Plin-26::egl-1(+)::NMDS 3’UTR] | Luc. | 11.4 (22/193) |
aAnimals were grown at 25˚C.
