# Supplementary figures and images for: Engineering threshold-based selection systems
Source: G3 (Bethesda). 2021 Jul 14;11(9):jkab234. doi: 10.1093/g3journal/jkab234 (PMC8496214; doi:10.1093/g3journal/jkab234)

## Slide 1
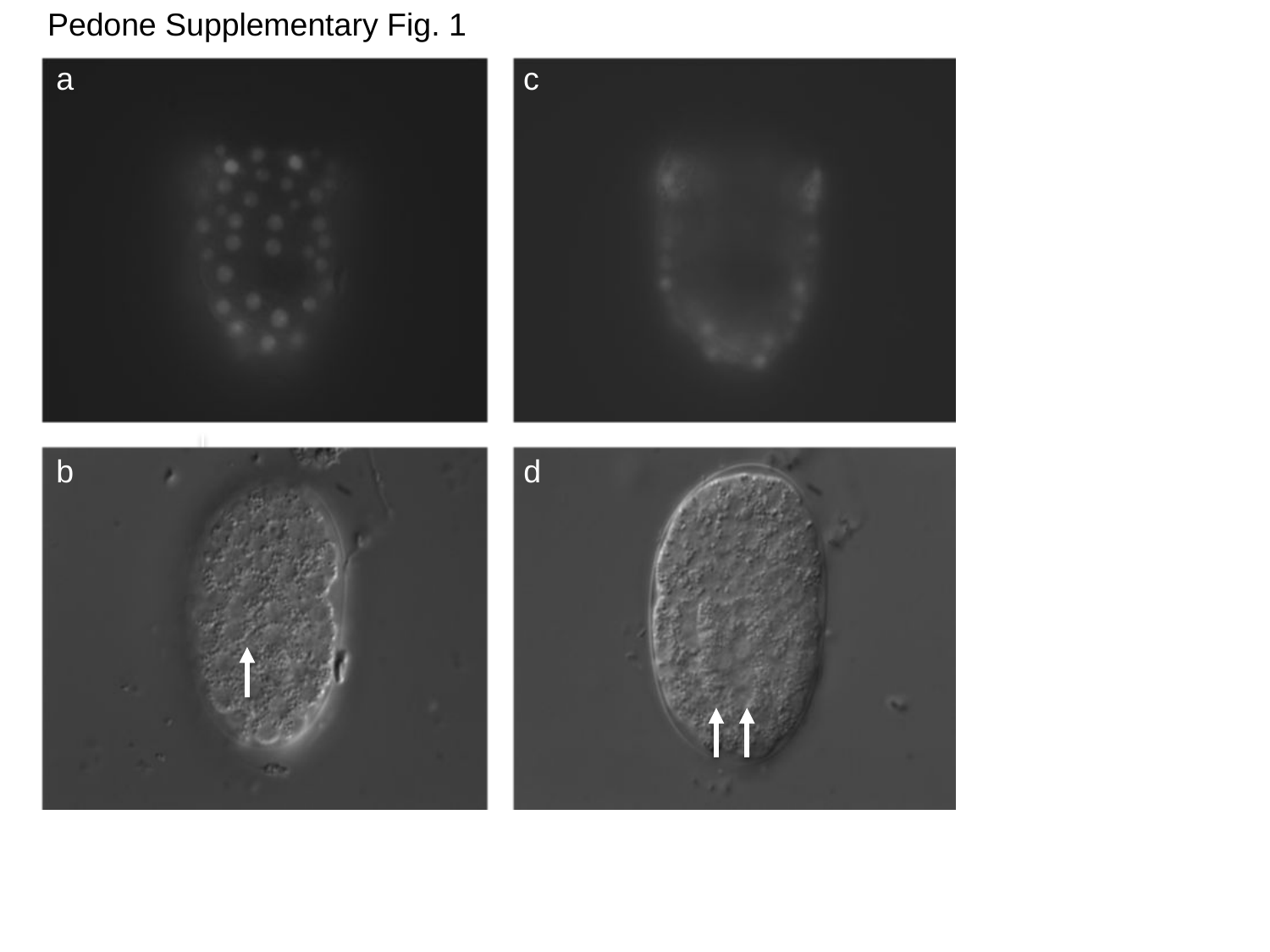

Pedone Supplementary Fig. 1
a
c
a
c
b
d
b
d

Supplement: jkab234_Supplementary_Data [file jkab234_supplementary_data.zip › jkab234-suppl_data/GENETICS-G3-2021-402425-s02.pptx]

## Slide 1
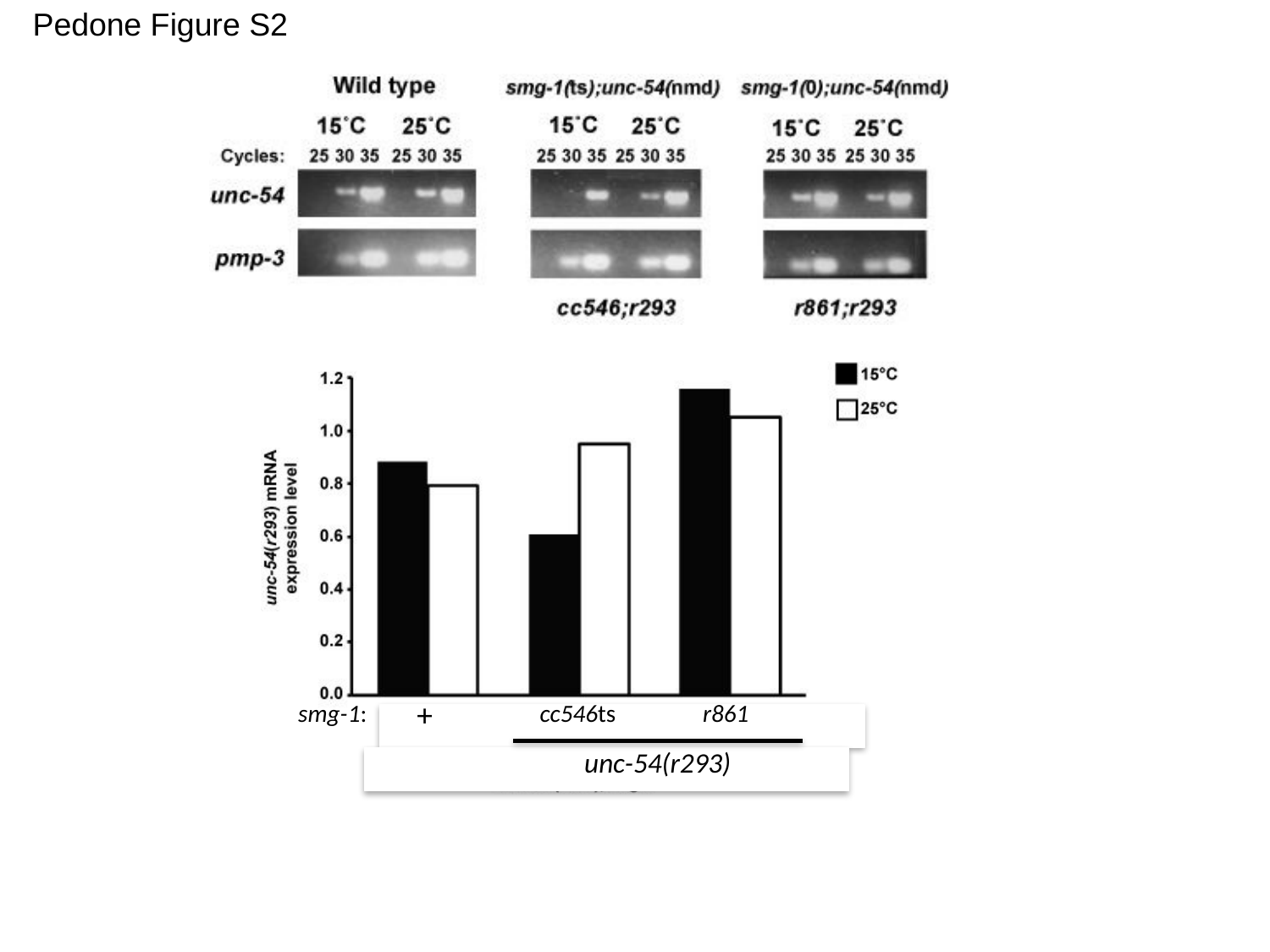

Pedone Figure S2
+
smg-1:
cc546ts
r861
unc-54(r293)

Supplement: jkab234_Supplementary_Data [file jkab234_supplementary_data.zip › jkab234-suppl_data/GENETICS-G3-2021-402425-s03.pptx]

## Slide 1
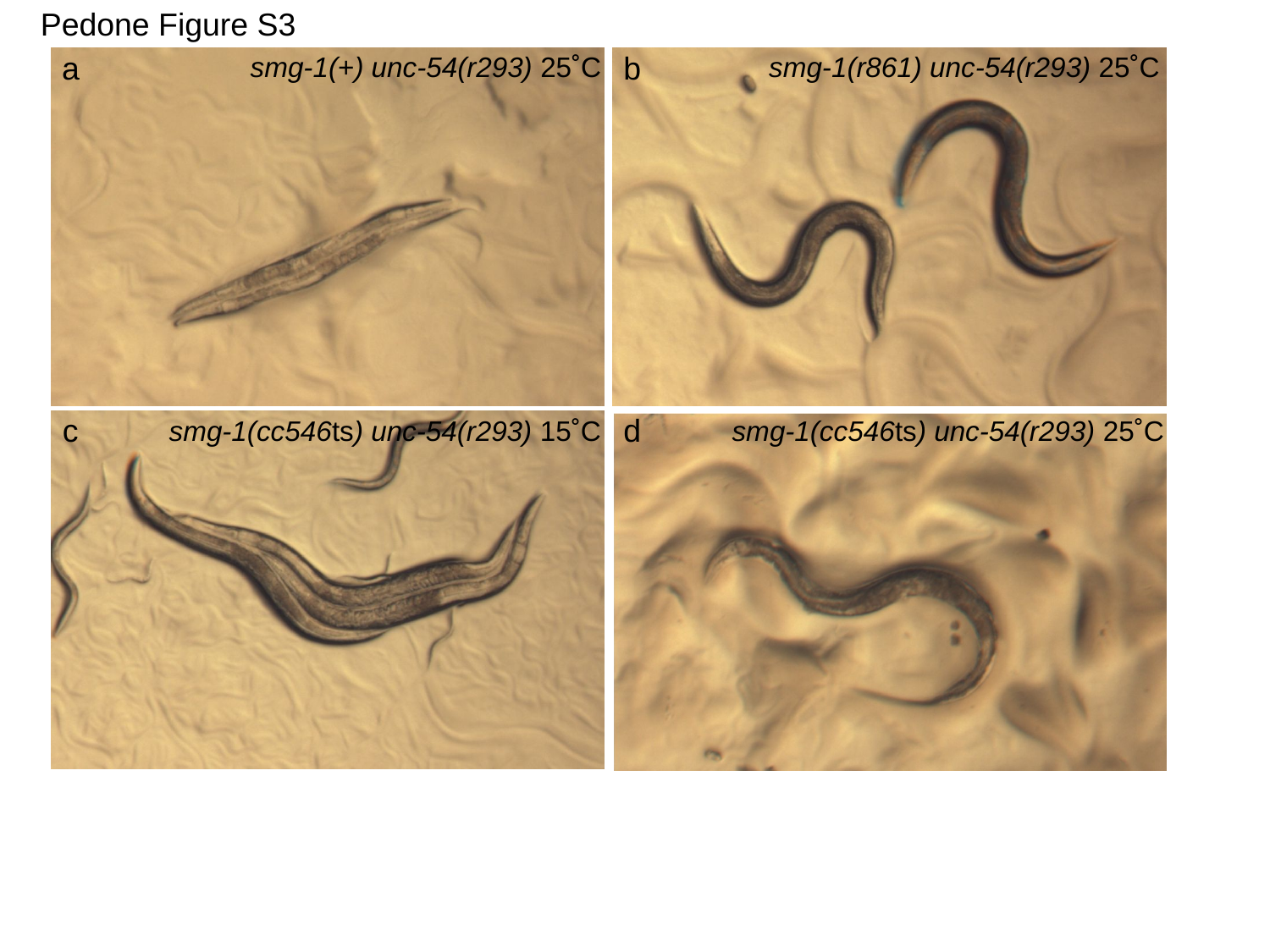

Pedone Figure S3
a
b
smg-1(+) unc-54(r293) 25˚C
smg-1(r861) unc-54(r293) 25˚C
c
d
smg-1(cc546ts) unc-54(r293) 15˚C
smg-1(cc546ts) unc-54(r293) 25˚C

Supplement: jkab234_Supplementary_Data [file jkab234_supplementary_data.zip › jkab234-suppl_data/GENETICS-G3-2021-402425-s04.pptx]

## Slide 1
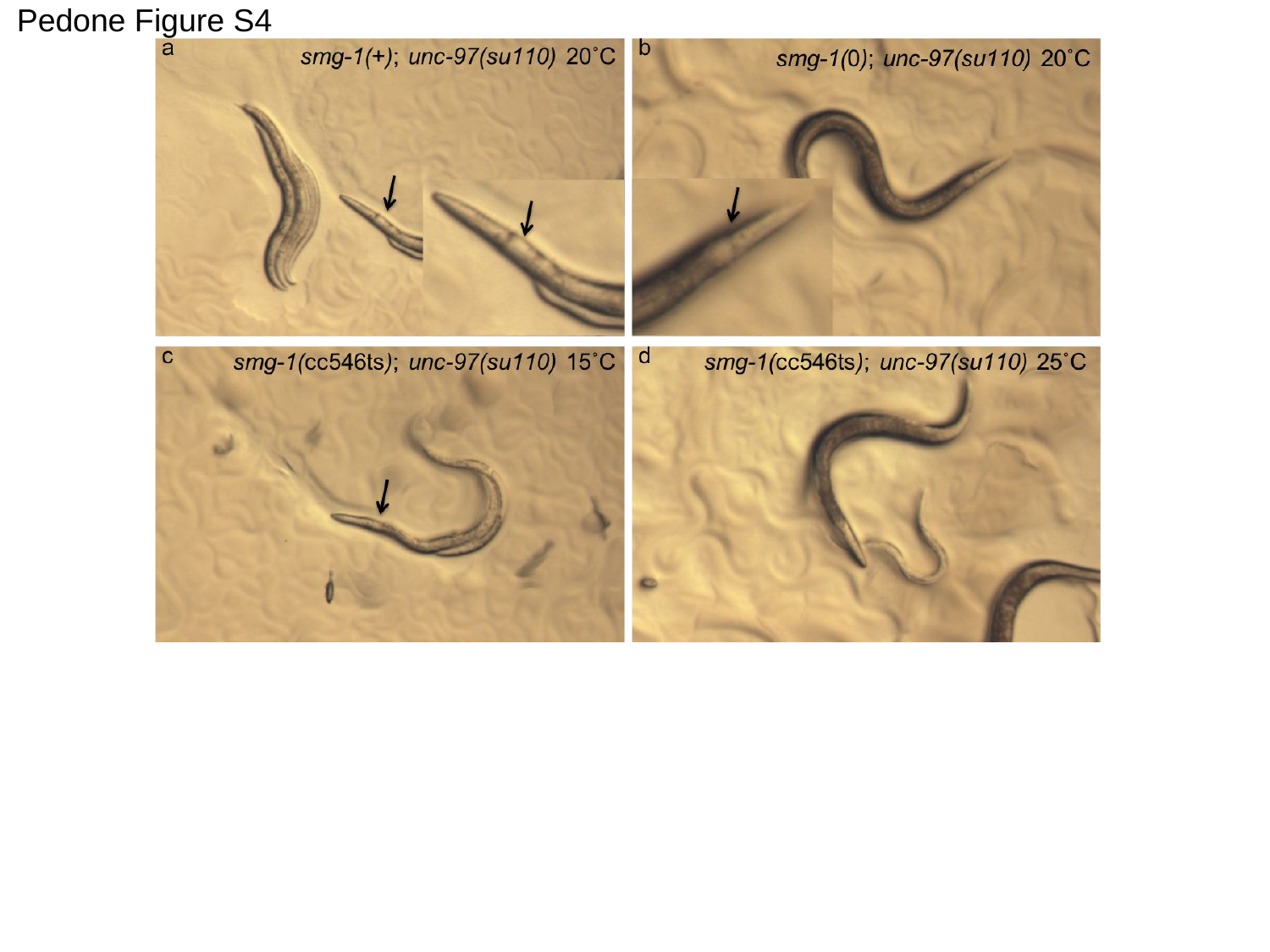

Pedone Figure S4

Supplement: jkab234_Supplementary_Data [file jkab234_supplementary_data.zip › jkab234-suppl_data/GENETICS-G3-2021-402425-s05.pptx]

## Slide 1
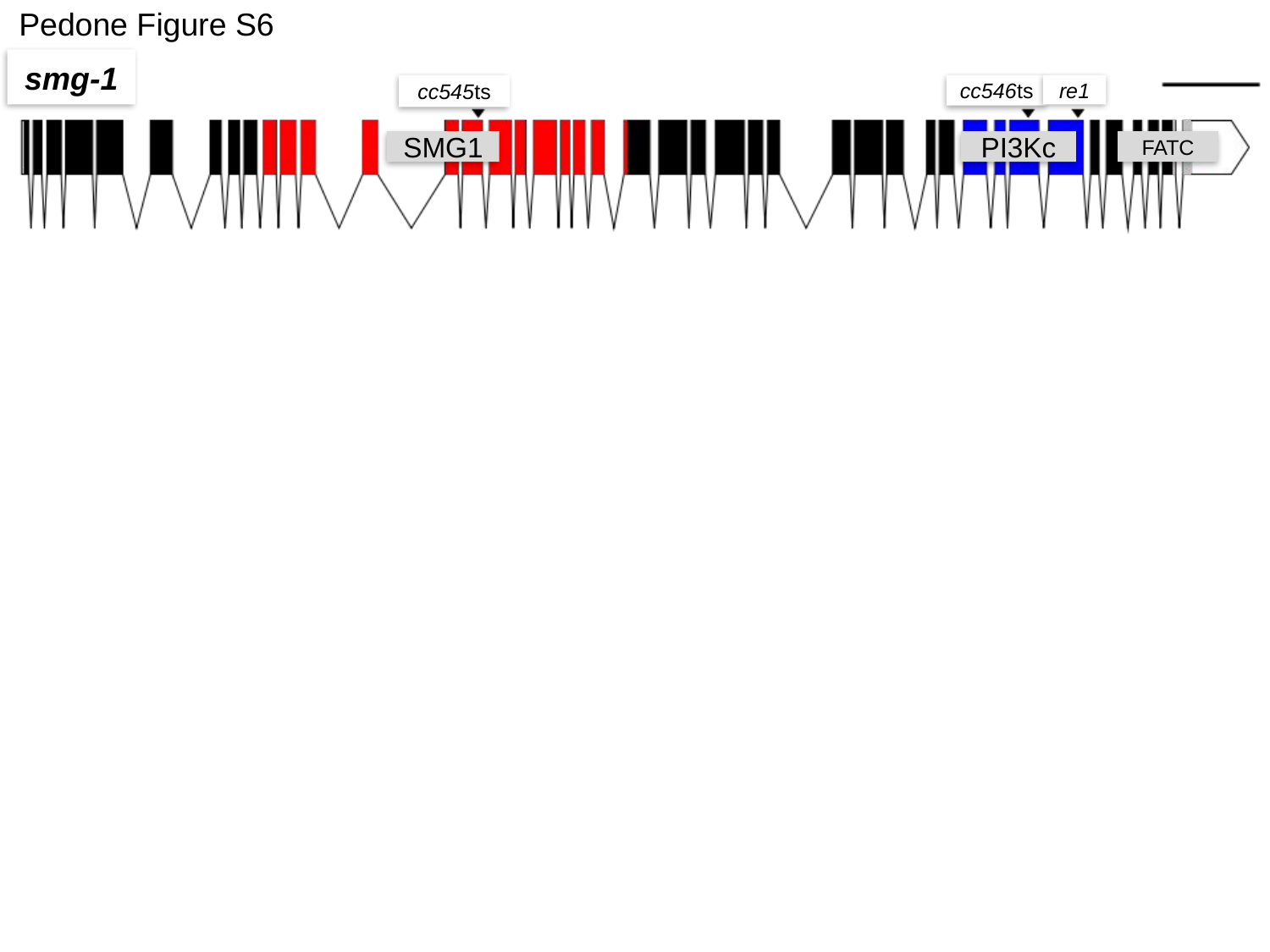

Pedone Figure S6
smg-1
cc545ts
cc546ts
re1
SMG1
PI3Kc
FATC

Supplement: jkab234_Supplementary_Data [file jkab234_supplementary_data.zip › jkab234-suppl_data/GENETICS-G3-2021-402425-s07.pptx]

## Slide 1
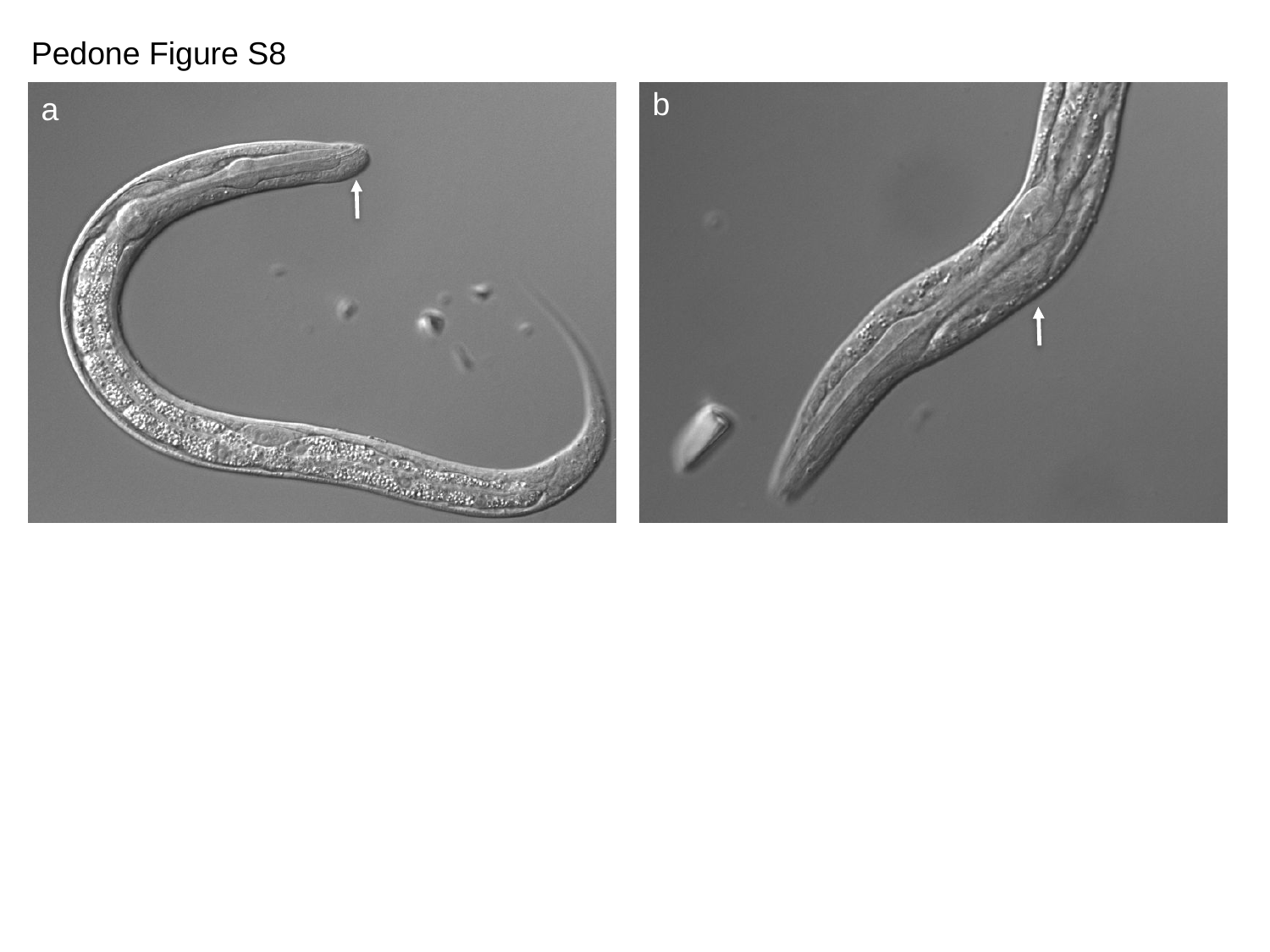

Pedone Figure S8
b
a

Supplement: jkab234_Supplementary_Data [file jkab234_supplementary_data.zip › jkab234-suppl_data/GENETICS-G3-2021-402425-s09.pptx]

## Slide 1
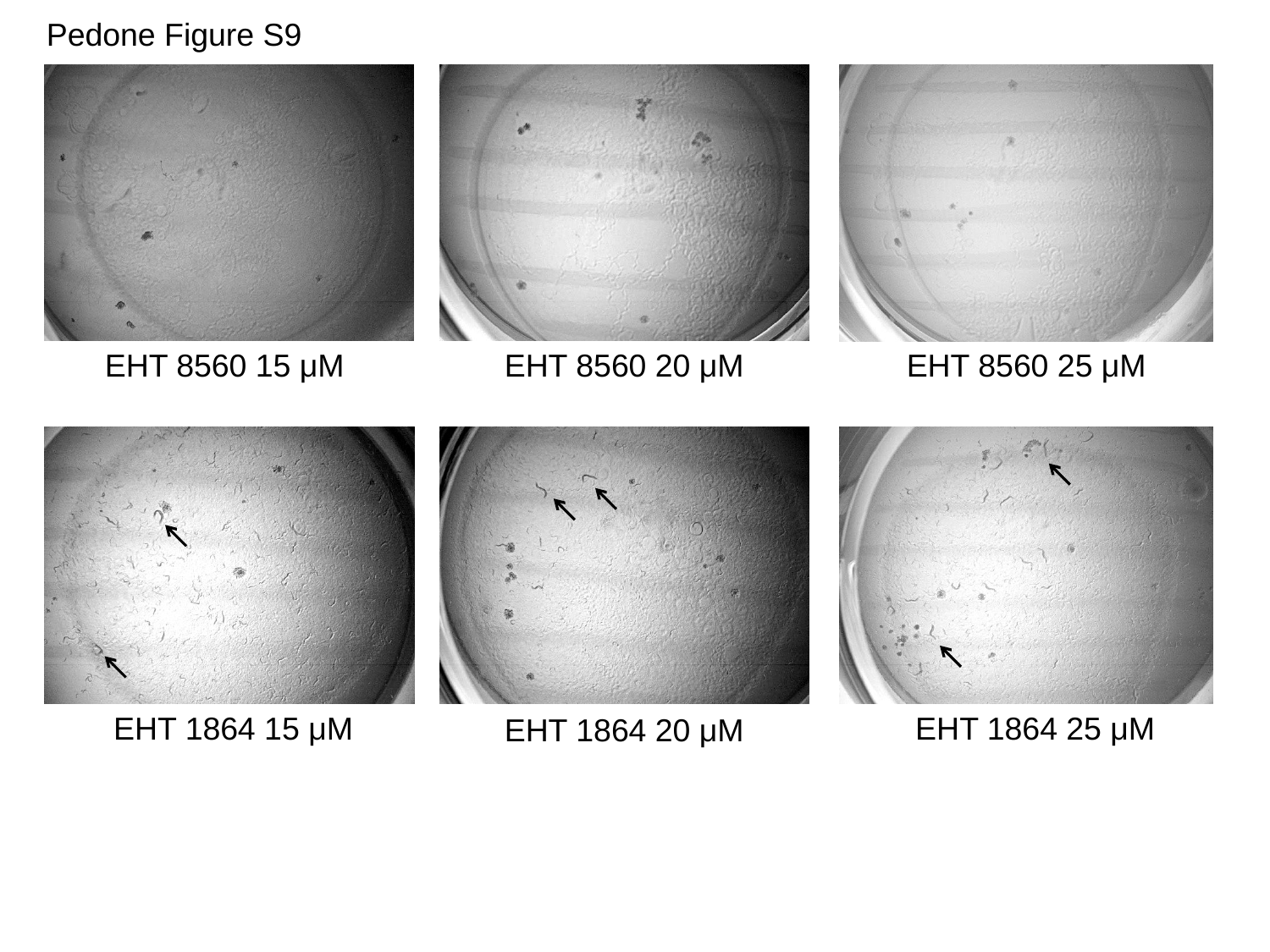

Pedone Figure S9
EHT 8560 15 μM
EHT 8560 20 μM
EHT 8560 25 μM
 EHT 1864 15 μM
 EHT 1864 25 μM
EHT 1864 20 μM

Supplement: jkab234_Supplementary_Data [file jkab234_supplementary_data.zip › jkab234-suppl_data/GENETICS-G3-2021-402425-s10.pptx]
